# Acetylation of FOXO1 activates Bim expression involved in CVB3 induced cardiomyocyte apoptosis

**DOI:** 10.1007/s10495-023-01924-3

**Published:** 2023-12-21

**Authors:** Yanan Hu, Lu Yi, Yeyi Yang, Zhixiang Wu, Min Kong, Zhijuan Kang, Zuocheng Yang

**Affiliations:** 1 Department of Pediatrics, Third Xiangya Hospital of Central South University, Changsha, Hunan 410013 People’s Republic of China; 2 Department of Medicine, Third Xiangya Hospital of Central South University, Changsha, Hunan 410013 People’s Republic of China

**Keywords:** Viral myocarditis, Coxsackievirus B3, FOXO1, Bim, Acetylation, HL-1 cell, Apoptosis

## Abstract

Viral myocarditis (VMC) is the major reason for sudden cardiac death among both children and young adults. Of these, coxsackievirus B3 (CVB3) is the most common causative agent of myocarditis. Recently, the role of signaling pathways in the pathogenesis of VMC has been evaluated in several studies, which has provided a new perspective on identifying potential therapeutic targets for this hitherto incurable disease. In the present study, in vivo and in vitro experiments showed that CVB3 infection leads to increased Bim expression and triggers apoptosis. In addition, by knocking down Bim using RNAi, we further confirmed the biological function of Bim in apoptosis induced by CVB3 infection. We additionally found that Bim and forkhead box O1 class (FOXO1) inhibition significantly increased the viability of CVB3-infected cells while blocking viral replication and viral release. Moreover, CVB3-induced Bim expression was directly dependent on FOXO1 acetylation, which is catalyzed by the co-regulation of CBP and SirTs. Furthermore, the acetylation of FOXO1 was an important step in Bim activation and apoptosis induced by CVB3 infection. The findings of this study suggest that CVB3 infection induces apoptosis through the FOXO1 acetylation-Bim pathway, thus providing new insights for developing potential therapeutic targets for enteroviral myocarditis.

**Supplementary Information:**

The online version contains supplementary material available at 10.1007/s10495-023-01924-3.

## Introduction

Viral myocarditis (VMC) is an inflammatory disease of the myocardium characterized by localized or diffused, acute or chronic lesions caused by a viral infection that can cause severe acute cardiac injury or chronic persistent cardiac injury. The incidence of VMC is 10–22 per 100,000 people, and the disease occurs predominantly in young and middle-aged patients, accounting for 12% of sudden deaths among patients < 40 years [1–3]. Coxsackievirus B3 (CVB3) is the leading cause of VMC, accounting for approximately 25% of VMC cases [4]. It is generally believed that viruses undergo rapid replication and induce direct damage to cardiomyocytes and via inflammatory factors [5]. To date, although animal, human myocardial biopsy, and in vitro studies have attempted to clarify the pathogenesis of VMC, the exact pathogenesis is still unclear. Moreover, prevention strategies and specific therapeutic drugs for the clinical treatment of VMC are lacking.

Several modes of cell death (such as apoptosis, autophagy, and ferroptosis) were identified to cause CVB3-induced cell death [6–9]. Among them, Bcl-2-interacting mediator of cell death (Bim), a proapoptotic BH3 domain-only member of the Bcl-2 family, has been found to potently bind to all anti-apoptotic Bcl-2 proteins with high affinity to trigger cell death; in addition, it is involved in the regulation of apoptosis in many different cell types [10–13]. Reportedly, Bim expression is tightly regulated by several pathways, such as Rb-E2F1 and the extracellular signal–regulated kinase/mitogen-activated protein kinase pathway. However, it is unclear whether Bim is involved in CVB3-induced VMC.

Recently, several studies have assessed FOXO family proteins and their interactions with Bim for inducing apoptosis in various cells [14–16]. The forkhead family of transcriptional regulators, FOXO transcription factors (FOXO1, FOXO3, FOXO4 and FOXO6), are involved in the regulation of the oxidative stress, cell cycle, apoptosis, DNA repair and immunomodulatory functions [17–19]. FOXO1 is currently considered a metabolic and antioxidant regulator that plays an important role in heart functioning. FOXO1 and FOXO3 are expressed in developing and adult cardiomyocytes, and specifically, FOXO proteins may play a role in regulating cardiomyocyte size via the induction of autophagy and inhibition of hypertrophy [20–22]. Mechanistically, FOXO proteins were appropriately post-translationally modified in response to phosphorylation or acetylation signals, thus activating the corresponding functions. These extracellular signals translate into activities that direct the regulation of proapoptotic genes, Bim and Fas Ligand [23, 24]. Therefore, it is essential to further elucidate how FOXO and its associated modifications are involved in apoptosis triggered by CVB3 infection.

Accordingly, the present study induced CVB3 infection in mice and cardiomyocyte HL-1 cells to establish in vivo animal models and in vitro cellular models for investigating the mechanism underlying CVB3-induced apoptosis. The results revealed that Bim played a key role in CVB3-induced apoptosis in VMC and that Bim expression was mediated by FOXO1. Furthermore, the mechanism underlying CVB3-induced FOXO1 phosphorylation and acetylation was evaluated in HL-1 cells. The findings confirmed that nuclear retention and co-regulation by CBP and SirTs family for FOXO1 acetylation were required for Bim activation.

## Materials and methods

### CVB3, cell culture and Infection

The CVB3 Nancy strain (Shanghai Jiao Tong University School of Medicine, China) was propagated in HeLa cells (Institute of Oncology, Central South University, China) and then stored at 80 °C. Virus titers were measured at the beginning of each experiment via the plaque assay. Cardiomyocyte HL-1 cells were purchased from Merck. These cell lines have been recently characterized and tested. HL-1 cells were seeded in 6-well plates one day before virus treatment. Then, the cells were incubated overnight in a medium containing 2% FBS for serum starvation. Subsequently, the cells were treated with CVB3 virus with an MOI of 10 (diluted with medium containing 2% FBS) at different time points. After incubation with the virus for 1 h, the cells were washed twice with phosphate-buffered saline, and the medium containing 2% FBS was added to the culture and incubated for 48 h. Subsequently, all cells were harvested for experiments. For the Sham group, infection was induced by adding the same volume of medium with 2% FBS instead of the virus. The cell culture supernatant was collected and stored at -80 °C for subsequent viral plaque assays. See *Supplementary Methods* for specific cell cultures.

### Animal models

Specific pathogen-free (SPF), male BALB/c mice (4 weeks, weight: 18–20 g) were obtained from the Department of Laboratory Animals of Central South University and were maintained under SPF conditions. After adaptive feeding for 1 week, mice were randomly assigned to different groups. The CVB3 group was intraperitoneally injected with 10^3^ TCID50 of the CVB3 virus (diluted to 100 µL with saline), and the Sham group was treated with the same volume of normal saline, as described previously [25]. Thereafter, the general appearance, behavior, and survival rate of the mice were observed daily. On days 1, 4, 7, and 14 post-infection, 6 mice were randomly selected from each group, and the surviving mice were sacrificed via cervical dislocation. Blood samples were collected for ELISA. Heart tissue from each group was collected for morphological, biochemical, and molecular biological analysis. Mice that died during or after treatment as well as mice in the CVB3 group without inflammatory cell infiltration in the myocardium were excluded from the analysis. All animal studies were approved by the experiment Animal Administrative Committee of Central South University (No.2021sydw0104). All animal experiments were in accordance with the relevant guidelines and regulations and followed the guidelines for the Care and Use of Laboratory Animals (Directive 2010/63/EU of the European Parliament). All efforts were made to reduce the suffering of the mice as much as possible.

### Hematoxylin and eosin (HE) staining

Heart tissue was fixed with 4% paraformaldehyde, prepared as wax blocks and cut into 5 μm sections. These sections were deparaffinized in xylene and dehydrated in alcohol. After staining, sections were sealed with neutral balsam and observed under an optical microscope.

### ELISA assay

ELISA assay was performed using the creatine kinase isoenzyme B (CK-MB) ELISA kit and cardiac troponin T (cTnT) ELISA Kit (JINGMEI Biotechnology, Jiangsu, China) according to the manufacturer’s instructions. Absorbance values were measured using EnVision Multilabel Plate Readers (PerkinElemer, MA, USA).

### Viral plaque assay

Viral plaque assay was performed as previously described [9] and in *Supplementary Methods*.

### Plasmid construction and cell transfection

The overexpression plasmids, negative control plasmids and small-interfering RNA (siRNA) were constructed by Tsingke Biotech (Changsha, China). HL-1 cells were seeded into six-well plates (1 × 10^5^ cells/well) and cultured for 24 h. Plasmids or siRNAs were transiently transfected using Lipofectamine 3000 reagent (Invitrogen, CA, USA) according to the manufacturer’s instructions. Western blotting was performed at 72 h after transfection. Related interference sequences are listed in Supplementary Table S1.

### Immunofluorescence (IF) assay

IF assay was performed according to the *Supplementary Methods*. The obtained paraffin sections or the prepared cell crawls were processed for IF staining. The antibodies in this study are provided in Supplementary Table S2.

### Cytotoxicity and cell proliferation

Cytotoxicity and cell proliferation were performed using the Calcein AM/PI Double Stain Kit (MKBio, Shanghai, China), and used according to the manufacturer’s instructions, as described in detail in *Supplementary Methods*.

### Cell apoptosis analysis

Apoptosis was determined by flow cytometry analysis of cells stained with FITC-Annexin V apoptosis detection kit (KeyGEN BioTECH, Jiangsu, China) by a Cytek Aurora spectral cytometer (Cytek Biosciences, Fremont, California, USA). At each time point, cells were harvested using Trypsin without EDTA (Beyotime, Shanghai, China). After centrifugation, cell pellets were washed and stained according to the manufacturer’s instructions and analyzed by FlowJo (Becton Dickinson, FKL, USA). The sum of the ratios of Annexin-V-positive (early apoptotic cells) and double-positive (late apoptotic or necrotic apoptotic) cells to the total number of analyzed cells reflects the apoptosis ratio.

### RNA extraction and quantitative real-time PCR (qRT-PCR) analysis

Total RNA from cells and tissues was extracted using TRIzol Reagent (Invitrogen). RNA concentration was measured using a NanoDrop ND-1000 spectrophotometer (Thermo Scientific, MA, USA). Complementary DNA was synthesized using the HiScript III RT SuperMix (Vazyme, Nanjing, China). qRT-PCR was performed using a LightCycler 480II Real-Time PCR instrument (Roche. Basel, Switzerland) for qRT-PCR. β-actin was used for normalization of the data. All primer sequences are listed in Supplementary Table S1.

### RNA sequencing

Collected mouse myocardial tissues were freshly frozen in liquid nitrogen and stored at -80 °C. The samples were sent to LC- Bio Technology Co., Ltd. (Hangzhou, Zhejiang, China) for RNA isolation, complementary DNA (cDNA) preparation, and DNA library preparation. The libraries were sequenced on an Illumina NovaSeq™ 6000.

### Western blotting

Western blotting was performed according to the *Supplementary Methods*. Myocardial tissue was placed in RIPA buffer (Beyotime) supplemented with protease inhibitor (Roche), phosphatase inhibitor (Roche) and phenylmethylsulfonyl fluoride using MagNA Lyser homogenizer (Roche) to grind the lysed tissue. The above operations were performed on ice. For cell, washed three times with phosphate-buffered saline and then lysed on ice with RIPA with a cocktail containing protease inhibitor and phosphatase inhibitor (Thermo Fisher, CA, USA). The antibodies in this study are provided in Supplementary Table S2.

### Subcellular fractionation assay

Subcellular fractionation assay was performed using the Protein Extraction Kit (Solarbio, Beijing, China), and used according to the manufacturer’s instructions, as described in detail in *Supplementary Methods*.

### Dual-luciferase reporter assay

Dual-luciferase reporter assay was performed using the Dual-Luciferase® Report (Promega, WI, USA), and used according to the manufacturer’s instructions, as described in detail in *Supplementary Methods*.

### Chromatin immunoprecipitation (ChIP) assay

ChIP assays were performed using the Pierce Magnetic ChIP kit (Thermo Fisher), and used according to the manufacturer’s instructions, as described in detail in Supplementary Methods. The primer sequences for promoter region are listed in Supplemental Table S1.

### Co-immunoprecipitation (Co-IP) assay

Co-IP assay was performed using the Pierce Classic Magnetic IP/Co-IP Kit (Thermo Fisher), and used according to the manufacturer’s instructions, as described in detail in *Supplementary Methods*. The antibodies in this study are provided in Supplementary Table S2.

### Statistical analysis

Experiments in this study were carried out with at least three replicates and all values are expressed as mean ± S.D. Statistical analyses were performed using SPSS (IBM SPSS 26.0, SPSS Inc). For normally distributed data, the differences between two groups were tested for statistical significance using an independent-sample two-tailed t-tests. For data that were normally distributed and when there was more than one group, one-way ANOVA was used, with Tukey’s comparison post hoc test. Where data were not normally distributed, Mann–Whitney U-tests were used to determine statistical significance. Values of *p <* 0.05 were considered statistically significant.

## Results

### Increased Bim expression in the heart of VMC mice

**Fig. 1:**
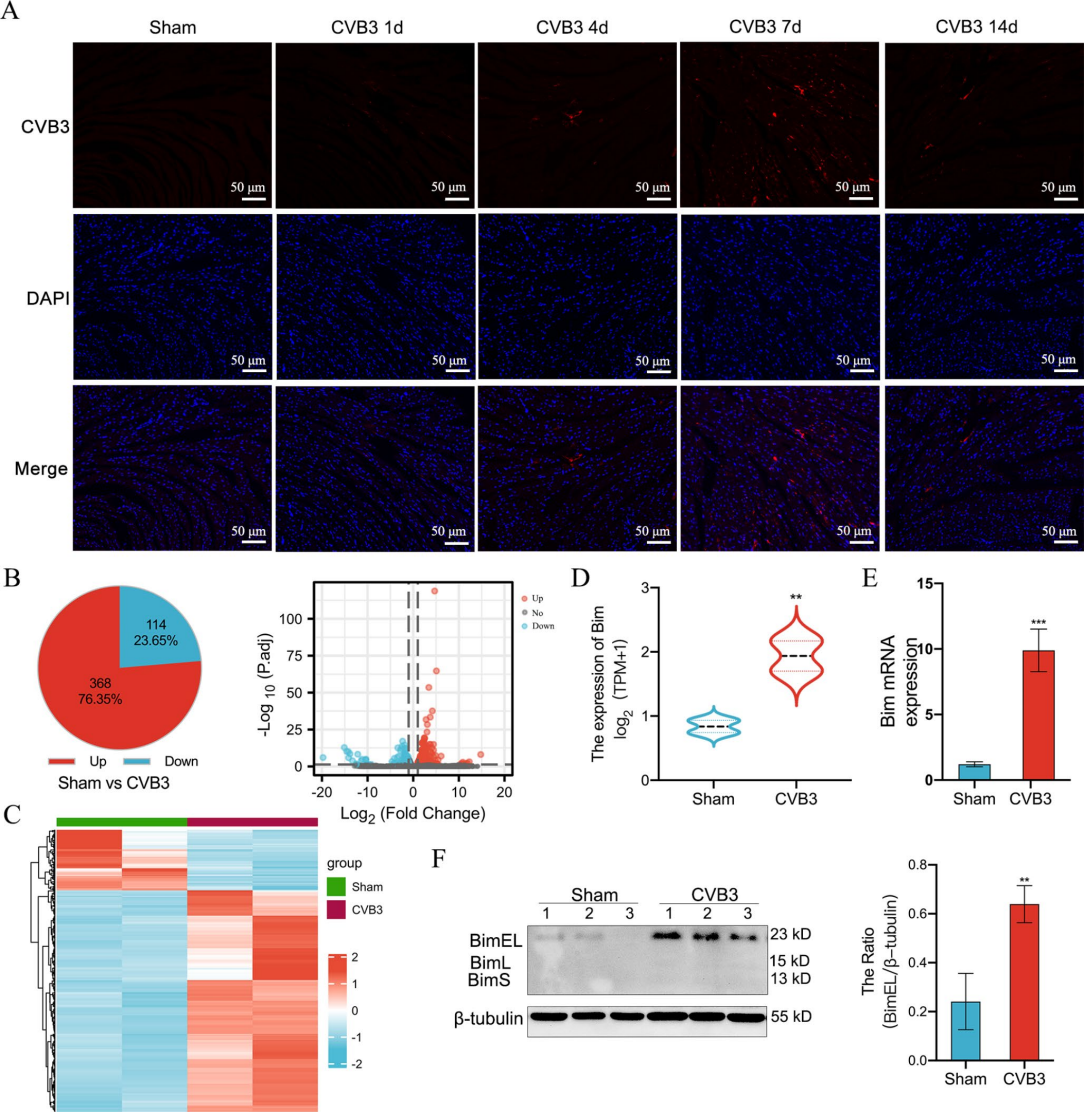
Increased expression of Bim observed in an in vivo model of CVB3-induced myocardial injury. (**A**) IF staining map of CVB3 (red) in mouse myocardial tissue in the Sham and CVB3 groups, blue staining indicates DAPI nuclear staining (scale bar = 50 μm); *n = 6*. (**B**) Pie and scatter plots of differentially expressed genes in the Sham and CVB3 groups after 7 days of CVB3 infection, with red indicating upregulated and blue indicating downregulated genes; these are positioned away from the unaltered genes (gray); *n = 2*. (**C**) Heat map of differentially expressed genes in the Sham and CVB3 groups; *n = 2*. (**D**) Sequencing results of differential mRNA expression of Bim; *n = 2*. (**E**) The mRNA level of Bim in the mice myocardium in the Sham and CVB3 groups were detected using qRT-PCR; *n = 3*. **(F)** Results of Bim protein levels in the Sham and CVB3 groups analyzed via western blotting; *n = 3*. ns: no statistical significance, *p < 0.05, **p < 0.01, ***p < 0.001

To investigate the mechanism underlying the effects of CVB3 in VMC, we first established the VMC model. The CVB3 group exhibited reduced activity, low food intake, weight loss, and shrugging and huddling; however, the symptoms gradually decreased after 9 days post-treatment. The changes in body weight are shown in Fig. S1A. Over time, compared with the Sham group, the CVB3 group demonstrated reduced heart size, uneven myocardial surface, and white membrane covering the heart, with greater edema of the heart by day 14 (Fig. S1B). Serum CK-MB and cTnT were higher in the CVB3 group than the Sham group at all time points (*p <* 0.05 for both) (Fig. S1C). The HE staining revealed progressive augmentation of inflammatory infiltration in the myocardial fibers and perivascular tissues over time in the CVB3 group (Fig. S1D). Furthermore, the IF assay of the myocardial tissue revealed that, unlike the Sham group, all CVB3 groups demonstrated CVB3 expression, with peak expression occurring at 7 days (Fig. 1A). These results indicate the successful establishment of the CVB3-induced VMC model, and 7 days performance is the most obvious.

In addition to directly causing structural destruction and functional metabolic damage to cardiomyocytes, viral infections can activate the Fas/FasL pathway, Bcl-2 family, and apoptotic protease (caspase) family to initiate cardiomyocyte apoptosis [26]. To further explore the mechanism by which CVB3 induces apoptosis in cardiomyocytes, we examined the changes in the expression of related genes after 7 days of CVB3 infection by RNA sequencing. For this, 482 differentially expressed genes were screened, among which 368 genes were found to be highly expressed and 114 were lowly expressed (|log2fc|>=1 & padj < 0.05) (Fig. 1B and C). Of the apoptosis-related genes, Bim was found to be significantly upregulated (Fig. 1D). We further performed RT-qPCR to detect the mRNA levels of Bim as well as western blotting to detect the protein levels of the three isoforms of Bim: BimEL, BimL, and BimS. The findings revealed a significant increase in both Bim mRNA and protein levels (BimEL) at days 7 and 14 in the CVB3 group compared with those in the Sham group, which was consistent with RNA-seq results (Fig. 1E and F).

### CVB3 induces apoptosis via Bim activation

**Fig. 2:**
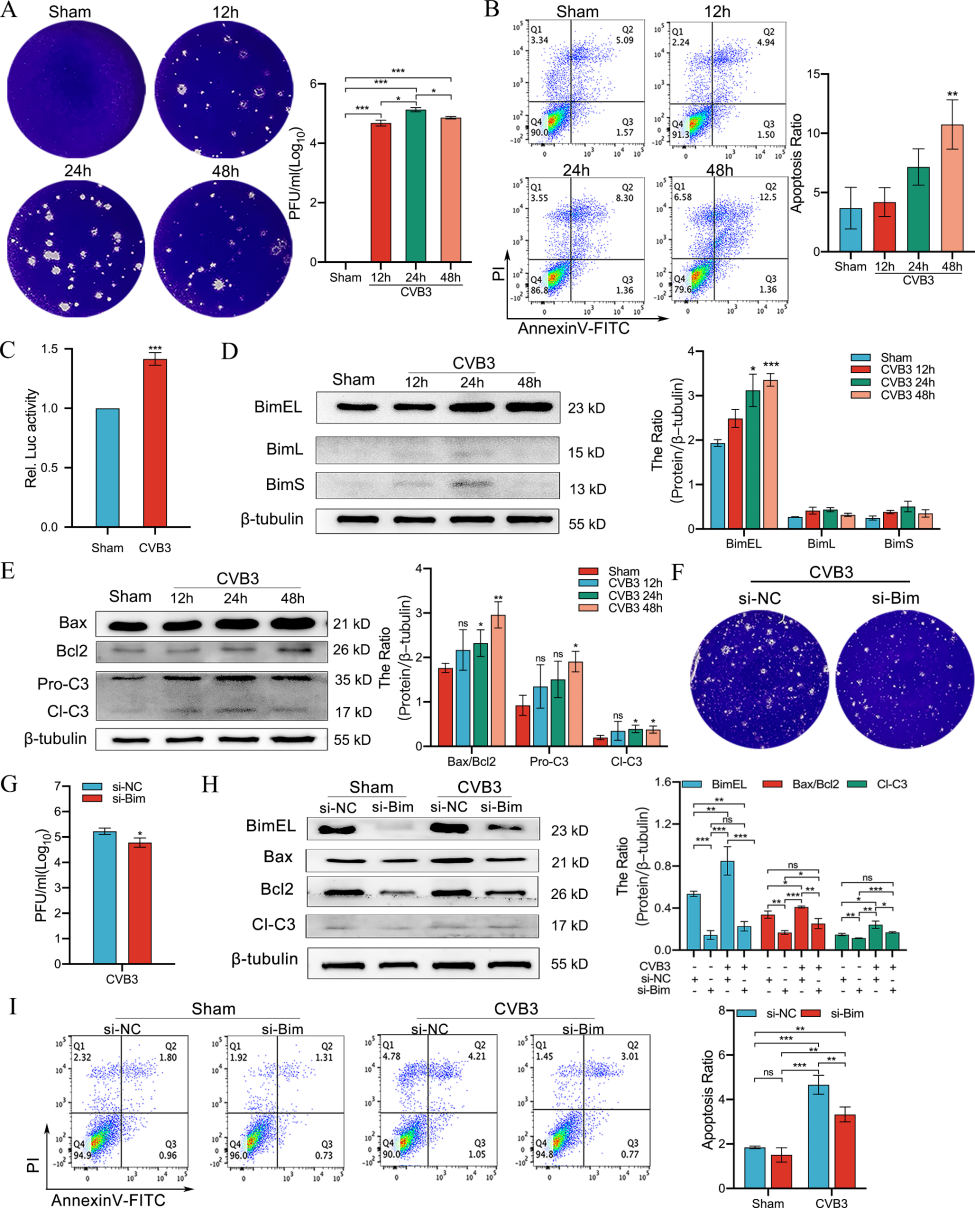
Changes in Bim expression in in vitro model of CVB3 infection in HL-1 cells. (**A**) Cultures of HL-1 cells were collected at 12, 24 and 48 h after CVB3 infection for viral plaque assay using Hela cells; *n = 3*. (**B**) Flow cytometry was used to detect apoptosis at 12, 24 and 48 h after CVB3 infection of HL-1 cells; *n = 3*. (**C**) Detection of Bim promoter activity after 48 h of CVB3 infection by dual-luciferase reporter assays; *n = 3*. (**D**) Changes in the expression of BimEL, BimL and BimS after CVB3 infection of HL-1 cells, as analyzed by western blotting; *n = 3*. (**E**) Changes in the expression of Bax/Bcl2 and cleaved-caspase3 proteins at 12, 24, and 48 h after CVB3 infection of HL-1 cells, as analyzed by western blotting; *n = 3*. (**F, G**) Cultures of HL-1 cells transfected with si-Bim were collected after CVB3 infection (48 h) for viral plaque assay using Hela cells; *n = 3*. (**H**) The expression of relevant proteins in HL-1 cells transfected with si-Bim after CVB3 infection (48 h), as analyzed using western blotting; *n = 3*. (**I**) Flow cytometry was performed to detect apoptosis in HL-1 cells transfected with si-Bim after CVB3 infection (48 h); *n = 3*. ns: no statistical significance, *p < 0.05, **p < 0.01, ***p < 0.001

To further explore the changes in Bim after CVB3 infection in mouse cardiomyocytes, HL-1 cells were used to construct an in vitro CVB3 infection model. Following pre-experiments, infection was induced at an MOI of 10. Viral plaque assay performed using supernatants collected at 12, 24 and 48 h post-infection demonstrated that CVB3 virus replication in HL-1 cells started at 12 h, with maximum viral replication occurring at 24 h (Fig. 2A). Assessment of viral cytotoxicity in HL-1 cells was determined using the Calcein AM/PI double-staining assay and revealed that significant dead cells were observed at 12 h post-infection and the number increased with time (Fig. S2). We further performed flow cytometry to determine the apoptotic effect of CVB3 infection in cardiomyocytes. As shown in Fig. 2B, the HL-1 cells in the CVB3 group demonstrated time-dependent apoptotic exacerbations with the prolongation of infection compared with the Sham group. These results suggest that the CVB3 could infect HL-1 cells and induce death in vitro, which was consistent with the findings of the in vivo study.

To determine whether CVB3 causes Bim upregulation through transcriptional activation, we first searched for the Bim promoter region in the NCBI database (http://www.ncbi.nlm.nih.gov/) and Ensembl database (http://useast.ensembl.org/index.html). The results revealed a segment of -1900 to + 350 bp from the transcription start site. The gene fragment was cloned into the pGL3-basic vector and CVB3 regulation was determined by measuring luciferase activity. Following reporter molecule activity assays, we found that the luciferase activity of reporter molecules driven by fragments − 1900 to + 350 was significantly increased following CVB3 infection for 48 h (Fig. 2C).

After CVB3 infection, western blotting was conducted to detect the protein levels of the three isoforms of Bim. As shown in Fig. 2D, only BimEL protein levels showed a time-dependent increase with the prolongation of CVB3 infection. In addition, the ratio of Bax/Bcl-2 and cleaved-caspase3 increased significantly with time-dependent tolerance under the same conditions (Fig. 2E). These results suggest that Bim plays a major role in CVB3-induced apoptosis in HL-1 cells.

To further confirm the direct association, HL-1 cells were transiently transfected with siRNA against Bim (si-Bim) or non-specific siRNA (si-NC), followed by CVB3 infection. First, viral plaque assay showed that si-Bim significantly reduced the release of viral progeny (Fig. 2F and G). Moreover, the number of dead cells in the si-NC group was significantly higher than that in the si-Bim group after CVB3 infection (Fig. S3). With regard to the levels of apoptotic proteins, the expression of Bax/Bcl-2 and cleaved-caspase3 was significantly lower in the si-Bim group after CVB3 infection than in the si-NC group (Fig. 2H). These observations were confirmed by flow cytometry (Fig. 2I). Together, these data suggest that CVB3-induced apoptosis in HL-1 cells is regulated by the pro-apoptotic protein Bim.

### FOXO1 regulates Bim transcription by binding to the Bim promoter region in CVB3 Infection

**Fig. 3:**
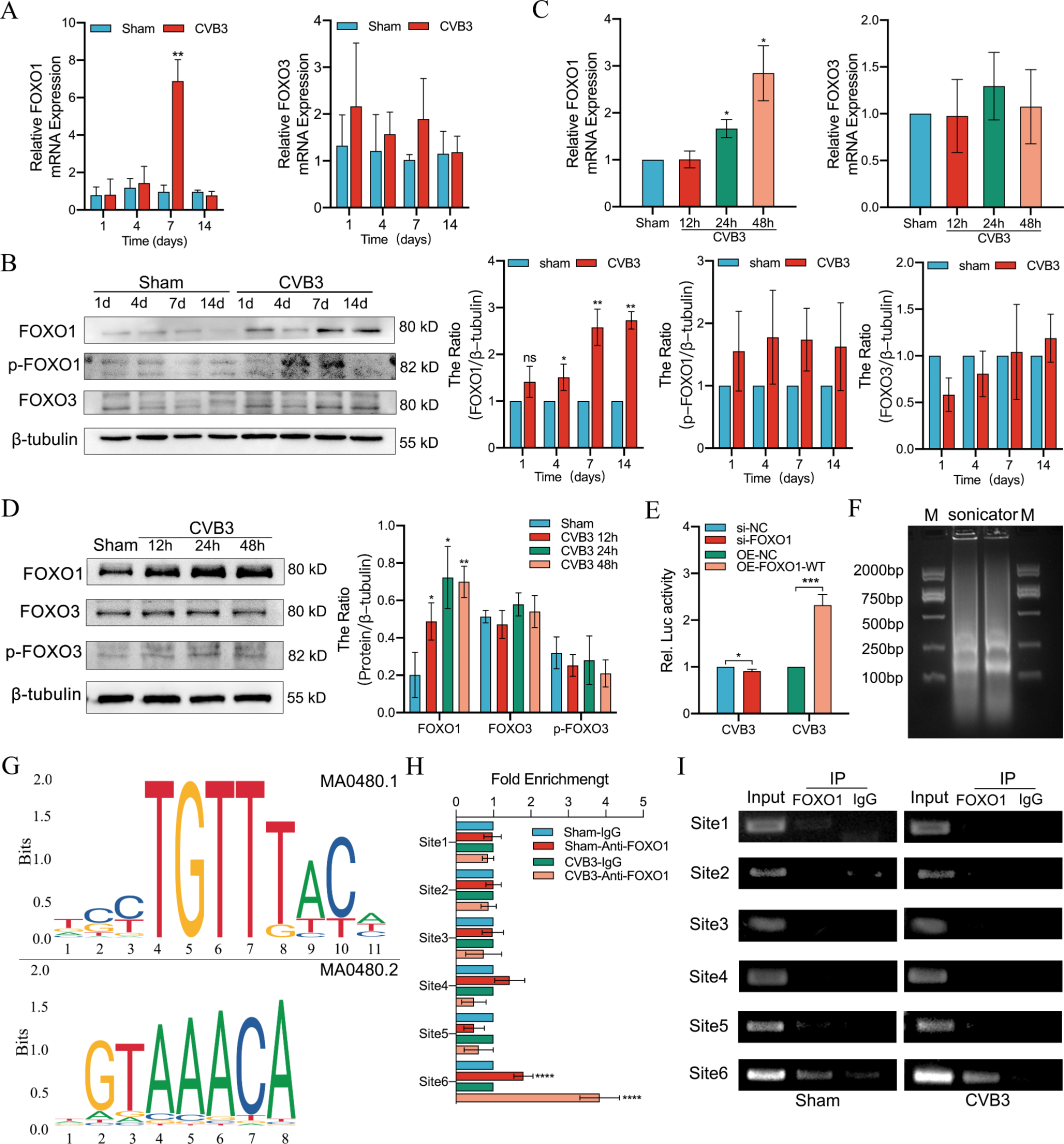
Activation of FOXO1-regulated Bim was involved in CVB3-infected myocarditis. (**A**) The mRNA levels of FOXO1 and FOXO3 in the mice myocardium in the Sham and CVB3 groups were detected using qRT-PCR; *n = 3*. (**B**) Changes in the expression of FOXO1, FOXO3 and p-FOXO1 in mice myocardium in the Sham and CVB3 groups were analyzed using western blotting; *n = 3*. (**C**) Detection of FOXO1 and FOXO3 mRNA levels by qRT-PCR at 12, 24 and 48 h after CVB3 infection in HL-1 cells; *n = 3*. (**D**) Changes in the expression of FOXO1, FOXO3 and p-FOXO1 at 12, 24 and 48 h after CVB3 infection in HL-1 cells, as analyzed using western blotting; *n = 3*. (**E**) Changes in Bim promoter activity after knockdown or overexpression of FOXO1 in HL-1 cells infected and uninfected with CVB3 (48 h), as detected using dual-luciferase reporter assays; *n = 3*. (**F**) Chromatin from HL-1 cell lysis buffer was subjected to agarose electrophoresis after sonication in the ChIP experiment; *n = 3*. (**G**) Transcription factor FOXO1 binding motifs from the JASPAR database. (**H**) ChIP-qPCR assay verified the direct binding of FOXO1 to the Bim promoter in HL-1 cells infected and uninfected with CVB3(48 h); *n = 3*. (**I**) Agarose electrophoresis was performed to further validate the ChIP-qPCR results; *n = 3*. ns: no statistical significance, *p < 0.05, **p < 0.01, ***p < 0.001, ****p < 0.0001

Bim mRNA levels were significantly upregulated in the myocardium of VMC mice (Fig. 1E), and the activity of the Bim promoter was significantly upregulated in the CVB3 group compared with that in the Sham group (*p* < 0.001) (Fig. 2C). To further explore the transcription factors that interact with the Bim promoter, we performed predictions using the UCSC database (https://genome.ucsc.edu/) and the PROMO database (https://alggen.lsi.upc.es/). Briefly, for the UCSC database, it was necessary to include the JASPAR component and then input the Bim promoter region for prediction to obtain 321 transcription factors; for the PROMO database, direct prediction for the Bim promoter region obtained 431 transcription factors. Intersecting the predictions from the two databases revealed 30 transcription factors, including the FOXO family (Supplementary Table S3). Therefore, the present study aimed to investigate whether CVB3-induced Bim expression is regulated by activating the FOXO family. The results revealed that the FOXO1 mRNA level was significantly increased at 7 days post-infection (*p* < 0.01), whereas the FOXO3 mRNA level showed no significant difference (Fig. 3A). Moreover, western blotting showed increased protein levels of FOXO1 and not FOXO3, in the CVB3 group at different times compared with the Sham group, with an upward trend (Fig. 3B). These results confirm that FOXO1 expression is significantly increased in the myocardial tissue of VMC mice.

Consistent with that observed in the VMC animal model, the FOXO1 mRNA and protein levels were significantly increased in HL-1 cells after CVB3 infection (*p* < 0.01). However, the levels for FOXO3 were not increased (Fig. 3C and D). This suggests that CVB3-infected HL-1 cells upregulated Bim expression through the transcription factor FOXO1, thus regulating the onset of apoptosis.

To further determine whether CVB3-induced Bim transcriptional activation is FOXO1-dependent, we evaluated the effect of FOXO1 on Bim promoter activity using the Dual-luciferase reporter assays. The analysis showed that wild-type overexpression of FOXO1 (OE-FOXO1-WT) enhanced Bim promoter activity (Fig. 3E), whereas the knockdown of FOXO1 decreased Bim promoter activity (Fig. 3E).

In addition, the binding sites of FOXO1 to the Bim promoter were predicted using the JASPAR database (http://jaspar.genereg.net/) (Fig. 3G). For this, six high-binding, fraction-binding sites (Site1-Site6) were assessed using the ChIP assay (Supplementary Table S1). The sheared DNA length (200–2000 bp) was confirmed using agarose gel electrophoresis (Fig. 3F). ChIP-qPCR analysis was performed in HL-1 cells using an anti-FOXO1 antibody, with rabbit IgG being the negative control. The results revealed that Bim binds to Site6 located at -11 to 0 and not to other regions in the non-infected and CVB3-infected cells (Fig. 3H and I). These results suggest that FOXO1 directly activates Bim transcription by binding to specific motifs on the Bim promoter.

### FOXO1/Bim axis regulates CVB3-induced cardiomyocyte apoptosis

**Fig. 4:**
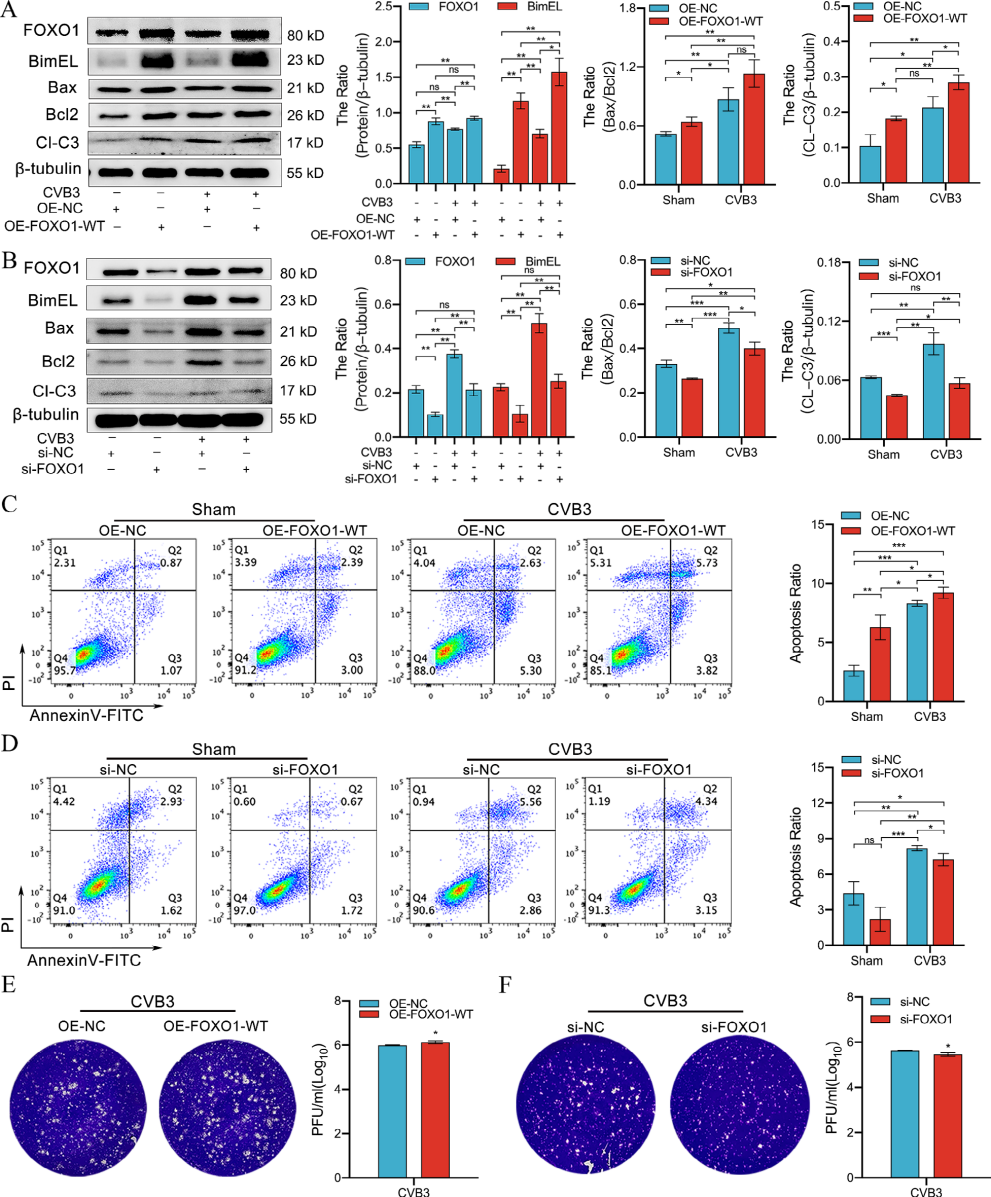
FOXO1 regulates apoptosis of CVB3-infected HL-1 cells. (**A**) HL-1 cells were infected with CVB3 (48 h) after transfection with OE-FOXO1-WT or OE-NC and were then harvested and subjected to western blot analysis to evaluate the expression of FOXO1, Bim, Bax/Bcl2, and cleaved-caspase3; *n = 3.* (**B**) After transfection of HL-1 cells with si-FOXO1 or si-NC, the cells were harvested under the same infection conditions and subjected to western blot analysis to identify the expression of FOXO1, Bim, Bax/Bcl2, and cleaved-caspase3; *n = 3.* (**C, D**) Flow cytometry was used to detect HL-1 apoptosis after CVB3 infection (48 h) with overexpressed or knocked down FOXO1; *n = 3.* (**E, F**) Cultures from the OE-NC group, OE-FOXO1- WT group, si-NC group, and si-FOXO1 group were collected 48 h after infection with CVB3, respectively, and viral plaque assay was performed using Hela cells; *n = 3.* ns: no statistical significance, *p < 0.05, **p < 0.01, ***p < 0.001

To verify that FOXO1-regulated Bim activation is involved in the onset of CVB3-induced apoptosis, HL-1 cells were transfected with the OE-FOXO1-WT and infected with CVB3 for 48 h. As shown in Fig. 4A, FOXO1 was significantly expressed after transfection than after Sham treatment. In addition, Bim expression was significantly increased in response to CVB3 infection (Fig. 4A), and the levels of the apoptotic proteins Bax/Bcl2, and cleaved-caspase3 were significantly increased. To further elucidate these results, HL-1 cells were transfected with si-FOXO1 or si-NC and then infected with CVB3 for 48 h. As shown in Fig. 4B, si-FOXO1 transfection significantly reduced FOXO1 and Bim protein levels in HL-1 cells. Moreover, Bim expression was significantly reduced after CVB3 infection, along with a significant reduction in the levels of apoptotic proteins Bax/Bcl2 and cleaved-caspase3 (*p* < 0.05). These results were confirmed by an apoptosis assay using flow cytometry (Fig. 4C and D), indicating that FOXO1 could regulate apoptosis in HL-1 cells infected by CVB3 via Bim activation.

Furthermore, the viral plaque assay was performed to detect the effect of FOXO1 on the viral replication of CVB3. The results revealed that the replication of the CVB3 virus was higher in the OE-FOXO1-WT group than in the non-specific plasmid (OE-NC) group. Moreover, it was significantly lower in the si-FOXO1 group than in the si-NC group (Fig. 4E and F). Notably, the number of dead cells in the OE-FOXO1-WT group was higher than that in the OE-NC group after CVB3 infection (Fig. S4), whereas the dead cells in the si-FOXO1 group were significantly lower than those in the si-NC group (Fig. S5). These results indicate that FOXO1 expression is a prerequisite for Bim and apoptosis induced by CVB3 infection and further affects viral self-replication.

### CVB3 Infection causes nuclear retention of FOXO1

**Fig. 5:**
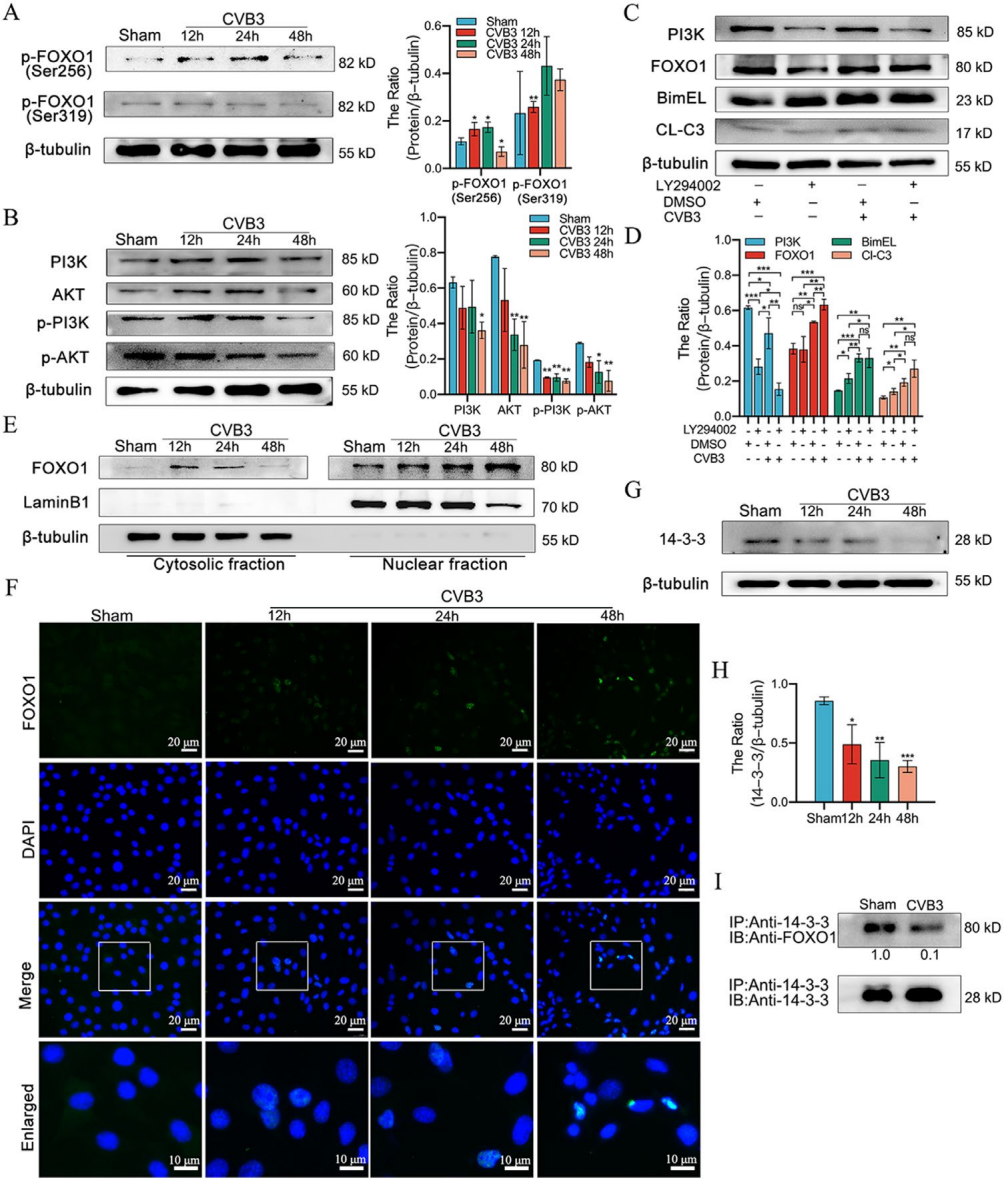
CVB3 infection fails to activate the PI3K/Akt cascade, which subsequently leads to nuclear retention of FOXO1. (**A**) HL-1 cells were infected with CVB3 for 12, 24, and 48 h and then harvested and subjected to western blotting to identify p-FOXO1 (Ser256) and p-FOXO1 (Ser319) expression; *n = 3.* (**B**) HL-1 cells were infected with CVB3 for 12, 24, and 48 h and then harvested and subjected to western blotting to identify PI3K, Akt, p-PI3K (Tyr 458), and p-Akt (Ser 473) expression; *n = 3.* (**C, D**) HL-1 cells were pretreated with LY294002 (50 µM) and then infected with CVB3 for 48 h. Cells were lysed and subjected to western blotting to evaluate PI3K, FOXO1, Bim, and cleaved-caspase3 expression; *n = 3.* (**E**) HL-1 cells were infected with CVB3-infected HL-1 cells for 12, 24, and 48 h, then nuclear and cytoplasmic proteins were isolated from the cells and immunoblot analysis was performed for FOXO1 protein using LaminB1 and β-tubulin as nuclear and cytoplasmic protein internal reference, respectively; *n = 3.* (**F**) To confirm the nuclear retention of FOXO1, CVB3-infected HL-1 cells were immunostained with FOXO1 antibody (green); blue indicates DAPI nuclear staining (scale bar = 20 μm). (**G, H**) HL-1 cells were infected with CVB3 for 12, 24, and 48 h and then harvested and subjected to western blot analysis to identify 14-3-3 protein expression; *n = 3.* (**I**) HL-1 cells were then infected with CVB3 for 48 h, and the proteins were extracted using anti-FOXO1 antibody, followed by Co-IP assay and protein immunoblot analysis with anti-14-3-3. The blots were additionally re-probed using FOXO1 antibody as loading controls; *n = 3.* ns: no statistical significance, *p < 0.05, **p < 0.01, ***p < 0.001

As the role of phosphorylation in the regulating of FOXO activity has been extensively characterized [27, 28], we next evaluated whether CVB3 infection resulted in changes in FOXO1 phosphorylation at the key sites Ser-256 and Ser-319. We found that p-FOXO1 (Ser-256) levels were significantly decreased at 48 h post-infection, whereas p-FOXO1 (Ser-319) levels showed no significant change (Fig. 5A). To examine whether the CVB3-induced increase in FOXO1 expression is regulated via the PI3K/Akt pathway, we infected HL-1 cells with CVB3 for 12, 24 and 48 h to detect the expression of PI3K and Akt. The results showed that CVB3 downregulated PI3K and Akt protein levels and inhibited PI3K and Akt phosphorylation (Fig. 5B). In addition, the expression of p-PI3K and p-Akt was decreased in the myocardial tissues of mice in the CVB3 group (Figure S6A). Although previous studies have found that growth factor-induced phosphorylation of FOXO proteins leads to their exclusion from the nucleus via Akt activation, our findings suggest that PI3K / Akt pathway activity is inhibited upon CVB3 infection, which may have reduced FOXO protein phosphorylation, resulting in nuclear retention and enhanced FOXO transcriptional activity. To further determine the role of the PI3K pathway associated with CVB3-mediated FOXO1 activation, HL-1 cells were pretreated with LY294002 (PI3K inhibitor) (50 µM) [29] and then infected with CVB3 for 48 h. Using LY294002 alone resulted in significant increase in the expression of Bim. However, compared with the cells infected with only CVB3, the cells treated with both LY294002 and CVB3 showed significantly increased expression of FOXO1 but no significant change in the expression of Bim (Fig. 5C and D). Moreover, CVB3 + LY294002 treatment increased cleaved-caspase3 shear compared with CVB3 infection alone (Fig. 5C and D). These results suggest that LY294002 inhibited PI3K and further promoted CVB3-induced apoptosis.

FOXO in the nucleus is involved in the transcription of Bim. Accordingly, we next determined whether FOXO accumulates in the nucleus. The results showed a steady increase of FOXO1 levels in the nuclear fraction of CVB3-infected cells (Fig. 5E). Furthermore, the intranuclear increase in FOXO1 was confirmed by IF, indicated by the enhanced staining of FOXO1 (green) in the nucleus (Fig. 5F). Previous studies have shown that once phosphorylated, FOXO1 binds to the 14-3-3 chaperone protein, which exports the complex from the nucleus, resulting in transcriptional downregulation of Bim [22]. To demonstrate this, HL-1 cells were infected with CVB3 and subjected to western blotting. The results revealed a progressive decrease in the expression of 14-3-3 chaperone proteins (Fig. 5G and H). To further clarify the interaction between 14-3-3 proteins, the Co-IP assay was conducted after CVB3 infection of HL-1 cells for 48 h. As expected, CVB3 infection greatly reduced the binding of 14-3-3 protein to FOXO1, indicating its retention in the nucleus (Fig. 5I). In addition, the IF assay in the myocardial tissues of mice in the CVB3 group showed enhanced staining for FOXO1 (green) along with reduced staining for 14-3-3 (red) (Fig. S6C).

### CVB3-induced FOXO1 acetylation is regulated by CBP and SirTs

**Fig. 6:**
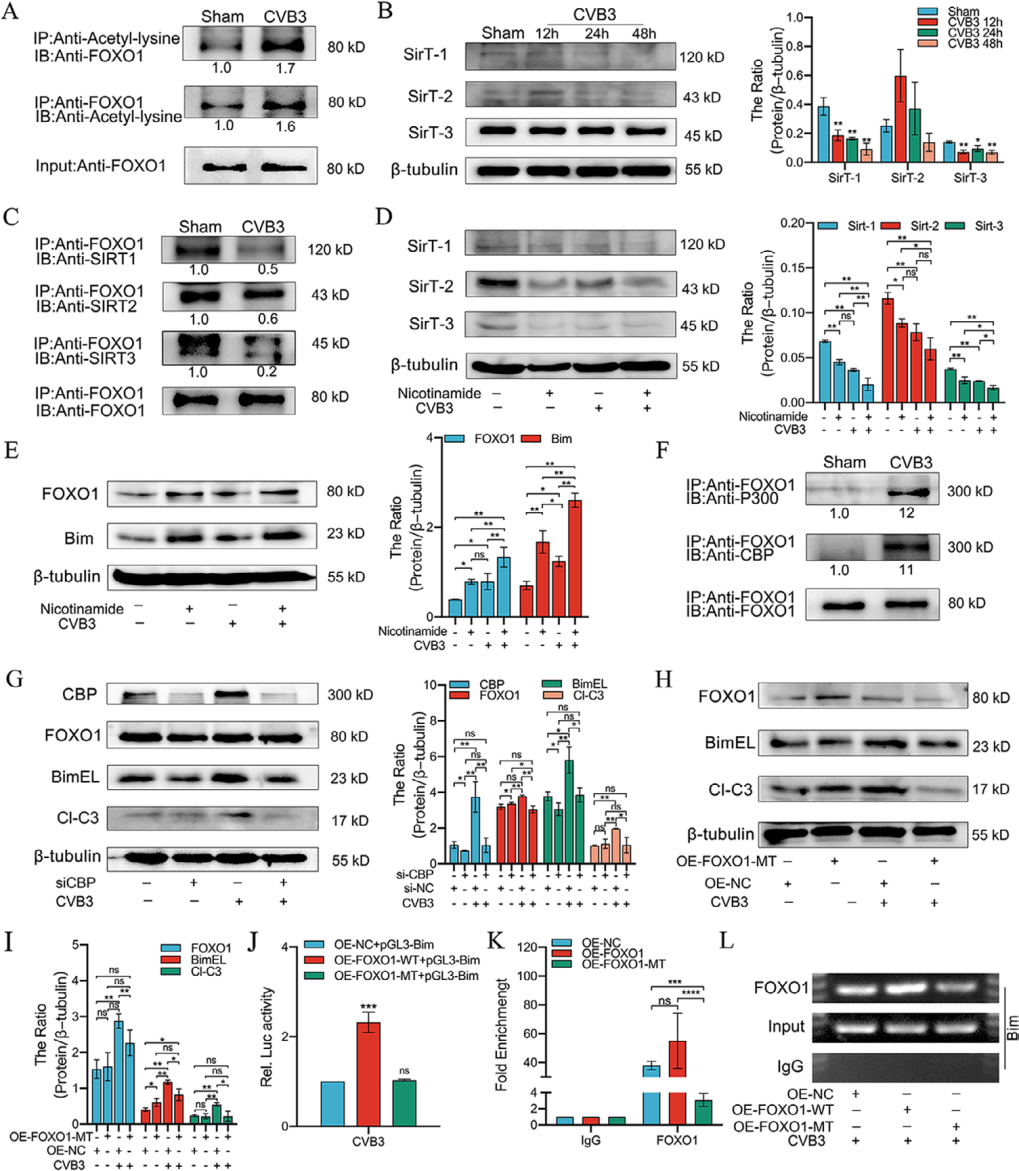
FOXO1 acetylation was regulated by CBP and SirTs and was necessary for CVB3-induced Bim expression. (**A**) HL-1 cells were infected with CVB3 for 48 h, and proteins were extracted for Co-IP using anti-acetyl-lysine antibody or anti-FOXO1, followed by western immunoblot analysis with anti-FoxO1 or anti-acetyl-lysine antibody. Before and after CVB3 infection, identical amounts of FoxO1 were used as a loading control, as shown as input; *n = 3.* (**B**) HL-1 cells were infected with CVB3 for 12, 24, and 48 h and were then harvested and subjected to western blotting to identify SirT-1, SirT-2, and SirT-3 expression; *n = 3*. (**C**) HL-1 cells were infected with CVB3 for 48 h, and proteins were extracted using anti-FOXO1 antibody for Co-IP assay, followed by western immunoblot analysis with anti-SirT-1, anti-SirT-2, and anti-SirT-3. The blots were additionally re-probed with FOXO1 antibody as a loading control; *n = 3.* (**D**) HL-1 cells were pretreated with nicotinamide (5 mmol/L) and then infected with CVB3 for 48 h. Cells were lysed and western blot analysis was performed to evaluate the expression of SirT-1, SirT-2, and SirT-3; *n = 3.* (**E**) HL-1 cells were pretreated with nicotinamide (5 mmol/L) and then infected with CVB3 for 48 h. Cells were lysed and western blotting was performed to identify the expression of FOXO1 and Bim; *n = 3.* (**F**) HL-1 cells were infected with CVB3 for 48 h and the proteins were extracted using anti-FOXO1 antibodies for Co-IP assay, followed by western immunoblot analysis with anti-CBP and anti-p300. The blots were additionally re-probed with FOXO1 antibody as a loading control; *n = 3.* (**G**) After transfection of HL-1 cells with si-CBP or si-NC, they were infected with CVB3 for 48 h and harvested and analyzed by western blotting to identify the expression of CBP, FOXO1, Bim, and cleaved-caspase3; *n = 3.* (**H, I**) After transfection of HL-1 cells with OE-FOXO1-MT or OE-NC, cells were infected with CVB3 for 48 h and harvested for western blotting to evaluate the expression of FOXO1, Bim, and cleaved-caspase3; *n = 3.* (**J**) Changes in Bim promoter activity after transfection with OE-FOXO1-WT and OE-FOXO1-MT were detected using dual-luciferase reporter assays; *n = 3*. (**K**) HL-1 cells were transfected with a FOXO1-WT or FOXO1-MT plasmid and were then infected with CVB3. After 48 h, cells were harvested for ChIP assay with a sequence of the Bim promoter using anti-FOXO1. The bands with anti-IgG served as negative controls; *n = 3*. (**L**) Agarose electrophoresis was performed to further validate the ChIP-qPCR results; *n = 3*. ns: no statistical significance, *p < 0.05, **p < 0.01, ***p < 0.001, ****p < 0.0001

Nuclear translocation induced by stress would trigger FOXO protein modification and regulation via acetylation/deacetylation. Using the Co-IP assay, we demonstrated that the acetylated lysine level of FOXO1 was significantly increased by CVB3 infection (Fig. 6A).

CBP/p300 and SirTs undergo acetylation or deacetylation following the acetylation of key lysine residues on FOXO, respectively [30]. To demonstrate this, HL-1 cells were infected with CVB3 and then subjected to western blotting. The results revealed a decrease in the expression of SirT-1 and SirT-3, the negative regulators of acetylation (Fig. 6B). Moreover, the expression of SirT-1, SirT-2, and SirT-3 was also decreased in the myocardial tissues of CVB3 mice (Figure S6B). To confirm whether SirTs were involved in FOXO1 acetylation by CVB3 infection, HL-1 cells were infected with CVB3 for 48 h, followed by immunoprecipitation with anti-FOXO1 antibody and immunoblotting with anti-SirT-1, anti-SirT-2 or anti-SirT-3. The results demonstrated that CVB3 reduced the recruitment of SirT-1, SirT-2, and SirT-3 to FOXO1 (Fig. 6C), which increased FOXO1 acetylation and promoted Bim expression. To confirm this hypothesis, HL-1 cells were treated with a strong inhibitor of SirTs, nicotinamide (5 mM) (Fig. 6D). The treatment increased CVB3-induced Bim expression (Fig. 6E), indicating that the inhibitory effect of nicotinamide on SirTs synergistically increased CVB3-induced Bim expression. Simultaneously, we noted a significant increase in FOXO1 levels in nicotinamide-treated cells (Fig. 6E). Together, these results suggest that SirTs are involved in regulating FOXO1 acetylation, leading to increased Bim expression.

To further determine the mechanism underlying CVB3-induced lysine acetylation, Co-IP assays were performed to test the interaction of coactivators (PCAF, CBP or p300) with FOXO1 (Fig. 6F). The results suggested that CVB3 infection recruited CBP and p300 to FOXO1 and induced the onset of acetylation. Notably, no protein interactions were noted between PCAF and FOXO1 (data not shown). Therefore, we further evaluated the effect of FOXO1 acetylation catalyzed by CBP on Bim expression in CVB3 infection. We used si-CBP to transfect HL-1 cells and then infect them with CVB3. The results revealed a significant reduction in CVB3 -induced Bim expression by si-CBP, demonstrating that CVB3 -induced Bim expression may occur by CBP-mediated FOXO1 acetylation (Fig. 6G). Moreover, si-CBP reduced the levels of cleaved-caspase3 compared with CVB3 infection alone (Fig. 6G). These results suggest that si-CBP inhibited FOXO1 acetylation and further decreased CVB3-induced apoptosis.

### FOXO1 acetylation is crucial for CVB3-induced bim expression

We next investigated the role of FOXO1 acetylation in the activation of Bim. For this, we used the mutant plasmid (FOXO1-MT), in which the CBP-dependent acetylation residue sites Lys-242, Lys-245 and Lys-262 were replaced with arginine. Cells were then transiently transfected with OE-FOXO1-MT or OE-NC, followed by infection with CVB3. As shown in Fig. 6H and I, CVB3 did not significantly upregulate mutant FOXO1 expression or upregulate Bim expression following CVB3 infection. To further confirm this, we performed a Dual luciferase reporter assay to analyze the transcriptional activity of FOXO1 on the Bim promoter. For this, HL-1 cells were transfected with OE-FOXO1-WT or OE-FOXO1-MT followed by a Bim-pGL3 luciferase reporter gene construct. After such treatment, cells were incubated in the presence or absence of CVB3 for 48 h and then evaluated for relative luciferase activity. A significant increase in relative luciferase activity of Bim was observed in cells transfected with OE-FOXO1-WT than in those treated with OE-NC (Fig. 6J). However, no significant increase in relative luciferase activity was observed in cells transfected with OE-FOXO1-MT (Fig. 6J). These data suggest that CVB3 induces Bim transcription via FOXO1 acetylation. This is consistent with the results of CVB3 infection-induced apoptosis in HL-1 cells (Fig. 6H and I), where CVB3 infection did not increase apoptosis in the OE-FOXO1-MT group, indicating that FOXO1 acetylation mediated by CBP recruitment is crucial for CVB3 infection to cause apoptosis.

**Fig. 7:**
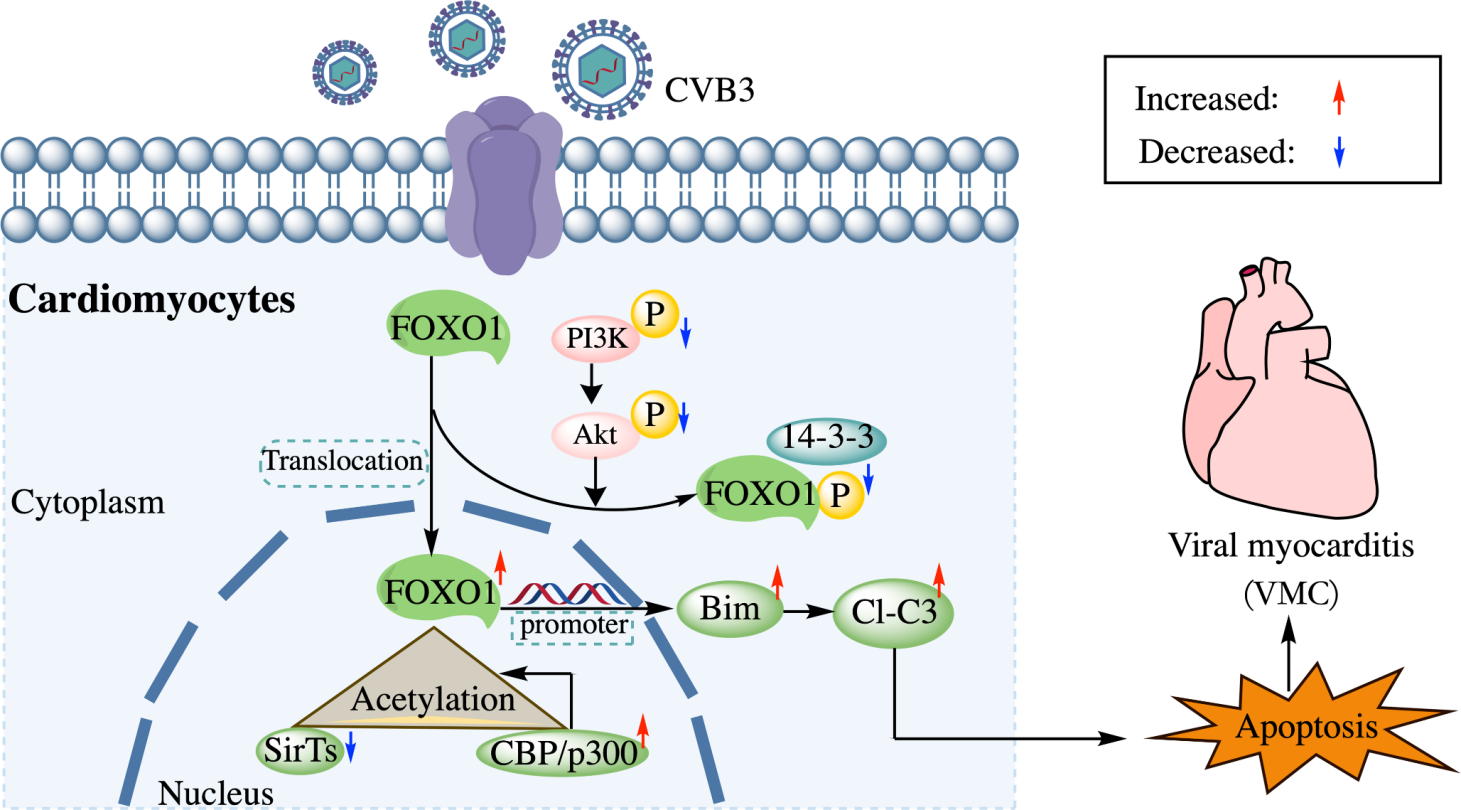
Schematic illustration of the mechanism by which the FOXO1 acetylation-Bim pathway in CVB3 infection promotes apoptosis

In addition, ChIP assays were used to identify whether acetylated FOXO1 binds directly to the Bim promoter. Figure 6 L shows that the binding of OE-FOXO1-WT to the Bim promoter is significantly increased following CVB3 infection. However, OE-FOXO1-MT was unable to bind to the Bim promoter (Fig. 6K and L). Thus, these findings suggest that CVB3 infection activates FOXO1 acetylation, while FOXO1 acts as a transcription factor to activate Bim, thus leading to increased apoptosis (Fig. 7).

## Discussion

In children, VMC is primarily induced by the virus CVB3. The virus induces cellular damage in the body as well as viral replication, which triggers uncontrolled inflammation even following viral clearance in the later stages of the disease [31]. Therefore, an effective strategy to treat VMC is necessary. Reportedly, the most important aspect of CVB3 infection is the onset of apoptosis in cardiomyocytes, which can be mediated via multiple pathways, including the activation of Bim [29, 32, 33]. However, the exact mechanism underlying this effect is unclear. The present study revealed a novel mechanism via which CVB3 can mediate apoptosis in cardiomyocytes: the FOXO1 acetylation-Bim pathway.

Apoptosis is one of the hallmarks of viral infection. In several models of infection, the apoptotic process is considered as a defense system, by which infected cells undergo cell death before the end of the viral life cycle, thereby limiting viral replication [34]. In these cases, nearby phagocytes eventually recognize and clear apoptotic vesicles. However, several viruses causing apoptosis may promote the release of viral progeny from infected cells, thereby increasing virus transmission and thus inducing disease progression [35, 36]. Therefore, although there is a growing interest in the activation of apoptotic pathways during CVB3 infection, many details regarding the involvement of CVB3 in the apoptotic process remain unclear [37]. Several pro-apoptotic pathways have been associated with the aberrant apoptosis of cardiomyocytes, with the extrinsic and intrinsic pathways being the major ones. The intrinsic pathway is regulated by both the pro-apoptotic (Bax, Bak, Bim and Bad) and anti-apoptotic (Bcl-2 and Bcl-xL) members of the Bcl-2 family [33, 38, 39]. Upregulation of Bim triggers cytochrome C dissociation from the mitochondria and contributes to apoptosome formation and caspase-3 activation. Although Bim is activated in the early stages of CVB3 infection in Hela cells, the extent of Bim expression and mechanisms underlying CVB3 infection of cardiomyocytes are poorly understood.

In the present study, increased Bim mRNA and protein levels were observed in in vivo and in vitro CVB3 infection models. To further confirm the function of Bim in CVB3 infection, we designed an siRNA and found that Bim knockdown reduced CVB3-induced apoptosis in HL-1 cells and decreased viral replication of CVB3. This suggests that Bim is a key pro-apoptotic integrator of CVB3-induced apoptosis in cardiomyocytes.

The current study additionally demonstrated the involvement of FOXO1 in Bim-regulated apoptosis triggered by CVB3 infection. FOXO1 siRNA reduced CVB3-induced apoptosis and decreased the viral replication of CVB3. Moreover, ChIP-qPCR and dual-luciferase reporter assay revealed that FOXO1 could directly bind to the Bim gene promoter, which clarified the direct regulation of Bim by FOXO1 at the transcriptional level in CVB3 infection.

We further explored the novel association of Bim activation with FOXO1 expression and post-translational modifications in CVB3 infection. Phosphorylation, ubiquitination and acetylation are the three major post-translational modifications of FOXO that regulate gene expression [28, 40, 41]. FOXO is a transcription factor that regulates the transcription of numerous target genes and undergoes nucleoplasmic shuttling in cardiomyocytes under different conditions [22, 42, 43]. The 14-3-3 protein plays a key role in FOXO nucleoplasmic shuttling, and 14-3-3 protein is a member of the highly conserved family of proteins that modulate intracellular signaling [44]. Phosphorylated Akt translocates to the nucleus from the cytoplasm [45] and phosphorylates FOXO1/3 [45, 46]. The phosphorylated FOXO demonstrates an increased binding to 14-3-3, leading to the translocation of FOXO1/3 from the nucleus to the cytoplasm, thus reducing its transcriptional activity [47–49]. In this study, we observed that CVB3 downregulated PI3K/Akt over time, reduced FOXO phosphorylation at 48 h (although there was an increase in p-FOXO1 during this period), and decreased binding to 14-3-3 chaperone proteins. Moreover, following CVB3 infection, nuclear retention of FOXO1 was demonstrated by IF and subcellular analysis in western blotting, which increased the expression of the pro-apoptotic gene Bim. This result was corroborated by the use of LY294002, a specific inhibitor of PI3K/Akt.

Stress-induced nuclear FOXO proteins bind to histone acetylase proteins, such as CBP/p300 and PCAF, resulting in increased acetylation of FOXO [30, 50, 51]. In addition, histone deacetylase proteins such as SirT-1 can interact with FOXO proteins and deacetylate them [51]. Consistent with these findings, the present study revealed two possible pathways for FOXO1 to be acetylated. First, FOXO1 might be acetylated via the direct recruitment of CBP to FOXO1. Our study provided some evidence to support that CVB3 infection promotes CBP involvement in FOXO1 acetylation. Moreover, si-CBP significantly reduced CVB3-induced Bim expression, further suggesting that this reduction is directed via CBP-mediated FOXO1 acetylation. Second, FOXO1 acetylation could be mediated by a weak interaction of the SirTs family with FOXO1. Consistently, our results also showed that CVB3 infection reduces the interaction of the SirTs family with FOXO1. Furthermore pretreatment of HL-1 cells with nicotinamide could ameliorate apoptosis caused by CVB3 infection. These findings suggest that CVB3 infection promotes the involvement of CBP and SirT-1 in the acetylation of FOXO1.

Currently, the role of acetylation in FOXO activity is still uncertain. Although the debate on acetylation-related activation is ongoing, the acetylation of FOXOs has been shown to have a positive effect on the induction of downstream gene expression [52–56]. In contrast, FOXO1 acetylation attenuates the transcriptional activity of FOXO1 [53, 54, 57]. The current study found that FOXO1 acetylation increased the response of Bim to CVB3 infection in HL-1 cells. Using acetylation-deficient mutants, our study further confirmed that FOXO1 mutated at the acetylation site exhibited a reduced ability to bind to the Bim promoter. In addition, the mutated FOXO1 demonstrated reduced apoptosis in response to CVB3 infection, indicating that apoptosis mediated by Bim in response to CVB3 infection can be regulated by FOXO1 acetylation.

In conclusion, our study identified FOXO1/Bim as a novel pathway that mediates cell death caused by CVB3 infection. Genetic modifications that inhibit FOXO1 and Bim prevent cell death and reduce viral replication. Mechanistically, nuclear retention caused by FOXO1 dephosphorylation, enhanced nuclear CBP/p300, and SirTs-mediated FOXO1 acetylation following CVB3 infection upregulate the expression of Bim and further trigger the onset of apoptosis. Based on these data, understanding the FOXO1 acetylation-Bim pathway in CVB3 infection may help to design effective strategies for the treatment of VMC.

### Electronic supplementary material

Below is the link to the electronic supplementary material.

Supplementary Material 1: Construction of a successful mouse model of VMC. (A) Body weight changes observed at different times in the Sham and CVB3 groups; *n = 6*. (B) The general morphology of the heart at different time points in the Sham and CVB3 groups; *n = 6*. (C) Comparison of serum myocarditis inflammatory markers CK-MB and cTnT at different time points in the Sham and CVB3 groups; *n = 6*. (D) Representative images of hematoxylin and eosin staining of mice myocardium in the Sham and CVB3 groups (scale bar = 50 μm); *n = 6*.

Supplementary Material 2: Cytotoxicity of CVB3 gradually increases over time. Calcein AM/PI double staining assay was used to assess the effect of CVB3 on HL-1 cells at 12, 24, and 48 h; green corresponds to calcein AM staining and represents live cells, and red corresponds to PI staining and represents dead cells (scale bar = 50 μm)

Supplementary Material 3: Knockdown of Bim improved the cytotoxicity of CVB3. Calcein AM/PI double staining assay was used to assess the cytotoxicity of CVB3 (48 h) to HL-1 cells after transfection with si-NC and si-Bim (scale bar = 20 μm)

Supplementary Material 4: FOXO1 overexpression enhanced CVB3 cytotoxicity. Calcein AM/PI double staining assay was used to assess the cytotoxicity of HL-1 cells after transfection with OE-NC and OE-FOXO1- WT at CVB3 infection (48 h) (scale bar = 50 μm)

Supplementary Material 5: FOXO1 knockdown induced CVB3 cytotoxicity. Calcein AM/PI double staining assay was used to assess the cytotoxicity of HL-1 cells after transfection with si-NC and si-FOXO1 at CVB3 infection (48 h) (scale bar = 50 μm)

Supplementary Material 6: Increased nuclear expression of FOXO1 in myocardial tissues of mice with VMC. (A) PI3K, Akt, p-PI3K (Tyr 458), p-Akt (Ser 473), and 14-3-3 protein levels in the Sham and CVB3 groups were analyzed using western blotting; *n = 3*. (B) The changes in the expression of CBP, SirT-1, SirT-2, and SirT-3 in mouse myocardium in the Sham and CVB3 groups were analyzed by western blotting; *n = 3*. (C) To confirm the expression of FOXO1 and 14-3-3, the myocardium of mice in the Sham and CVB3 groups was subjected to IF staining using the FOXO1 antibody (green) and the 14-3-3 antibody (red); blue indicates DAPI nuclear staining (scale bar = 100 μm)

Supplementary Material 7

Supplementary Material 8

## Data Availability

The datasets generated during and/or analyzed during the current study are available from the corresponding author on reasonable request.
