# Supplementary figures and images for: Acetylation of FOXO1 activates Bim expression involved in CVB3 induced cardiomyocyte apoptosis
Source: Apoptosis. 2023 Dec 21;29(7-8):1271–87. doi: 10.1007/s10495-023-01924-3 (PMC11263423; doi:10.1007/s10495-023-01924-3)

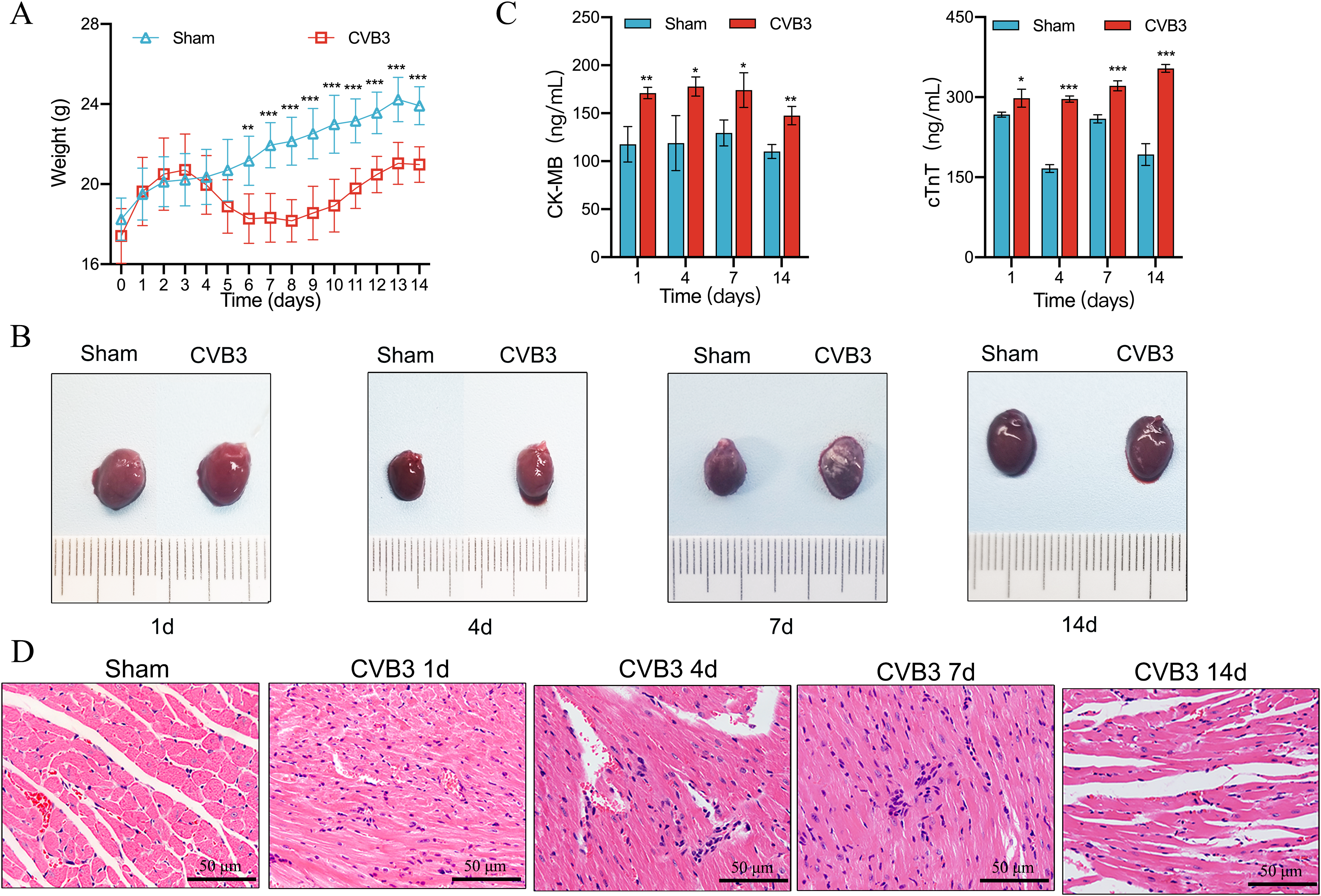

Supplement: Supplementary file 1 — Supplementary Material 1: Construction of a successful mouse model of VMC. (A) Body weight changes observed at different times in the Sham and CVB3 groups; n = 6. (B) The general morphology of the heart at different time points in the Sham and CVB3 groups; n = 6. (C) Comparison of serum myocarditis inflammatory markers CK-MB and cTnT at different time points in the Sham and CVB3 groups; n = 6. (D) Representative images of hematoxylin and eosin staining of mice myocardium in the Sham and CVB3 groups (scale bar = 50 μm); n = 6. [file 10495_2023_1924_MOESM1_ESM.png]

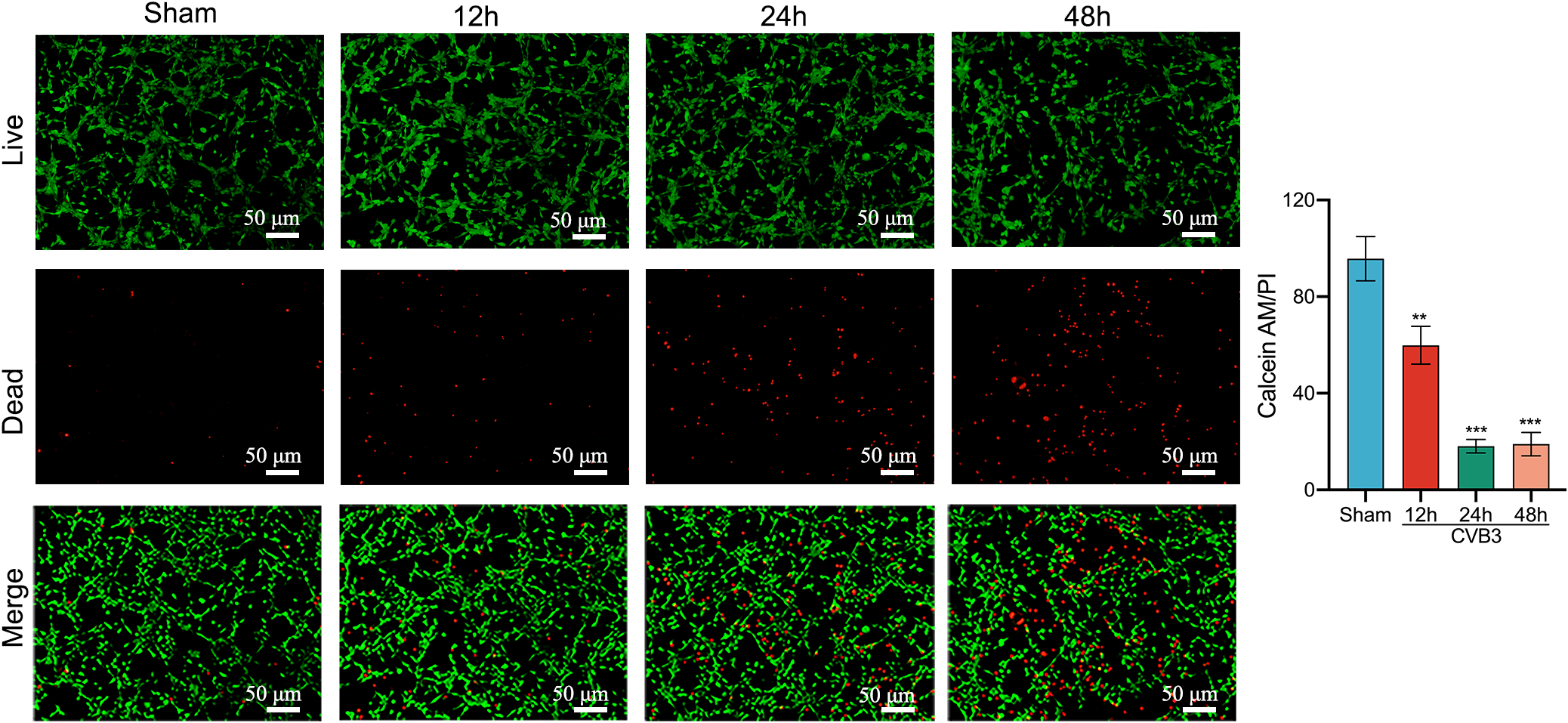

Supplement: Supplementary file 2 — Supplementary Material 2: Cytotoxicity of CVB3 gradually increases over time. Calcein AM/PI double staining assay was used to assess the effect of CVB3 on HL-1 cells at 12, 24, and 48 h; green corresponds to calcein AM staining and represents live cells, and red corresponds to PI staining and represents dead cells (scale bar = 50 μm) [file 10495_2023_1924_MOESM2_ESM.png]

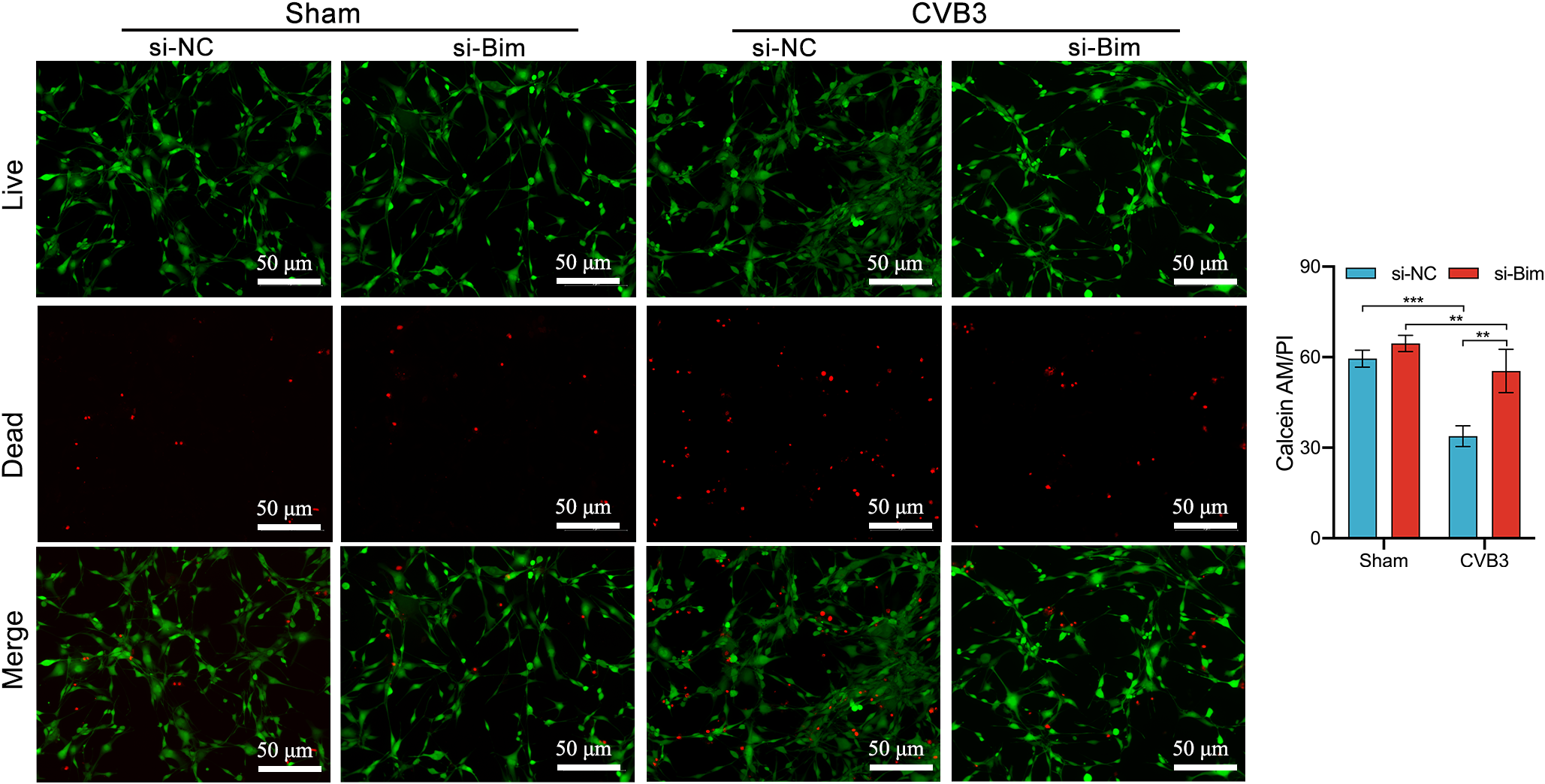

Supplement: Supplementary file 3 — Supplementary Material 3: Knockdown of Bim improved the cytotoxicity of CVB3. Calcein AM/PI double staining assay was used to assess the cytotoxicity of CVB3 (48 h) to HL-1 cells after transfection with si-NC and si-Bim (scale bar = 20 μm) [file 10495_2023_1924_MOESM3_ESM.png]

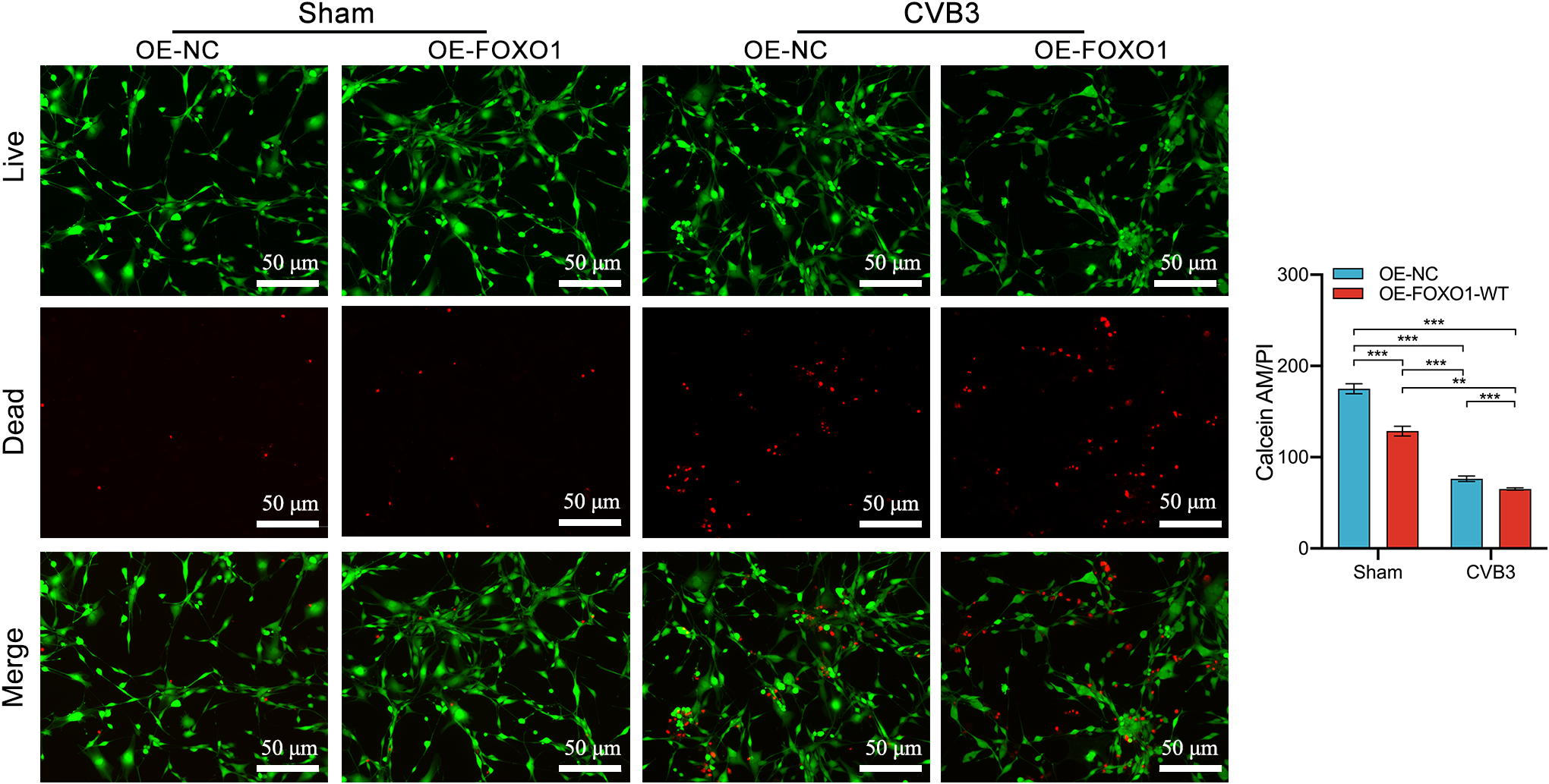

Supplement: Supplementary file 4 — Supplementary Material 4: FOXO1 overexpression enhanced CVB3 cytotoxicity. Calcein AM/PI double staining assay was used to assess the cytotoxicity of HL-1 cells after transfection with OE-NC and OE-FOXO1- WT at CVB3 infection (48 h) (scale bar = 50 μm) [file 10495_2023_1924_MOESM4_ESM.png]

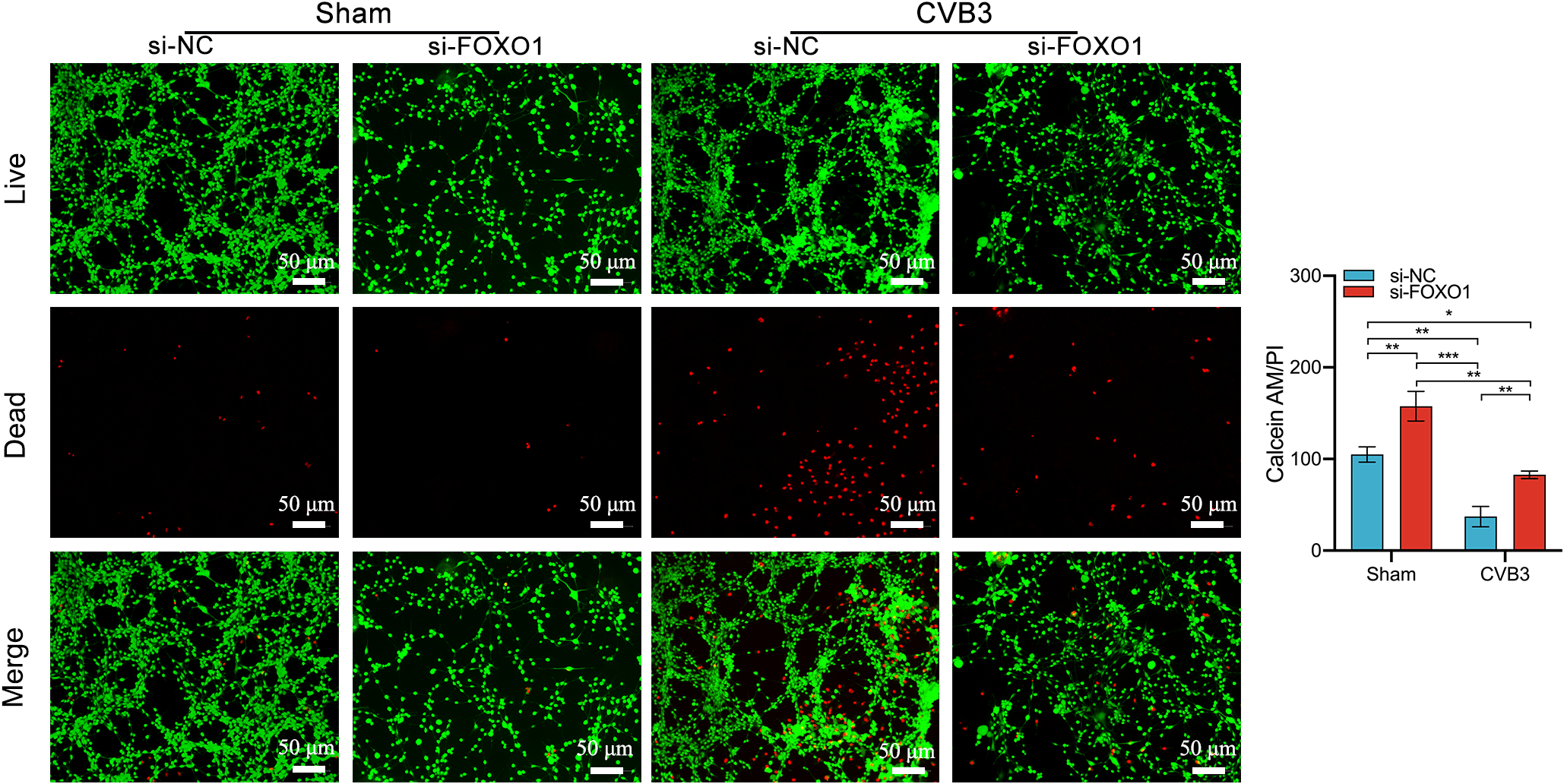

Supplement: Supplementary file 5 — Supplementary Material 5: FOXO1 knockdown induced CVB3 cytotoxicity. Calcein AM/PI double staining assay was used to assess the cytotoxicity of HL-1 cells after transfection with si-NC and si-FOXO1 at CVB3 infection (48 h) (scale bar = 50 μm) [file 10495_2023_1924_MOESM5_ESM.png]

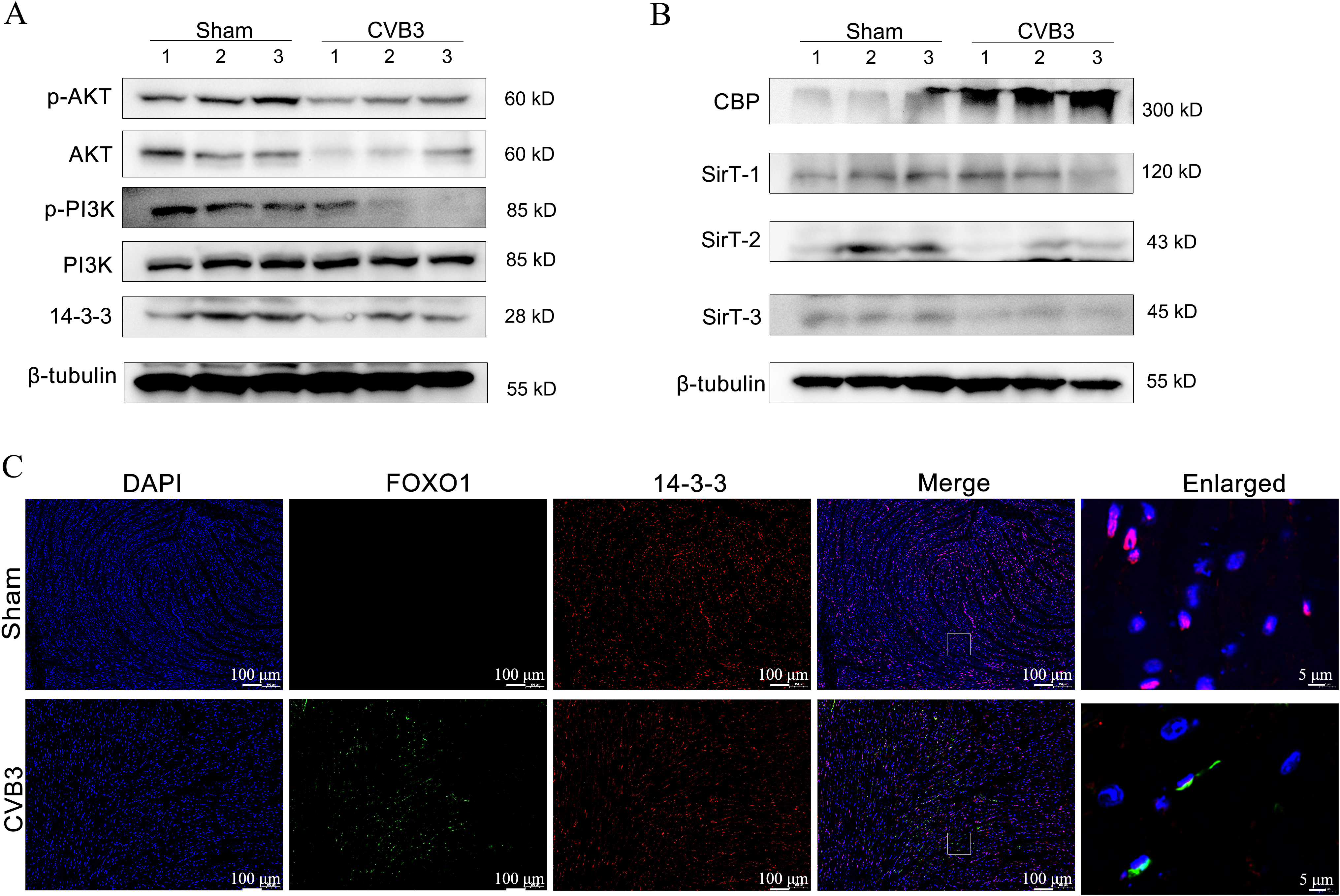

Supplement: Supplementary file 6 — Supplementary Material 6: Increased nuclear expression of FOXO1 in myocardial tissues of mice with VMC. (A) PI3K, Akt, p-PI3K (Tyr 458), p-Akt (Ser 473), and 14-3-3 protein levels in the Sham and CVB3 groups were analyzed using western blotting; n = 3. (B) The changes in the expression of CBP, SirT-1, SirT-2, and SirT-3 in mouse myocardium in the Sham and CVB3 groups were analyzed by western blotting; n = 3. (C) To confirm the expression of FOXO1 and 14-3-3, the myocardium of mice in the Sham and CVB3 groups was subjected to IF staining using the FOXO1 antibody (green) and the 14-3-3 antibody (red); blue indicates DAPI nuclear staining (scale bar = 100 μm) [file 10495_2023_1924_MOESM6_ESM.png]
