# Supplementary material for: Acetylation of FOXO1 activates Bim expression involved in CVB3 induced cardiomyocyte apoptosis
Source: Apoptosis. 2023 Dec 21;29(7-8):1271–87. doi: 10.1007/s10495-023-01924-3 (PMC11263423; doi:10.1007/s10495-023-01924-3)
Supplement: Supplementary file 8 — Supplementary Material 8 [file 10495_2023_1924_MOESM8_ESM.docx]

**Acetylation of FOXO1 Activates Bim Expression Involved in CVB3 Induced Cardiomyocyte Apoptosis**

Hu Yanan^1^, Yi Lu^1^, Yang Yeyi^2^, Wu Zhixiang^1^, Kong Min^1^, Kang Zhijuan^1^, Yang Zuocheng (corresponding author)^1^

^1^Department of Pediatrics, Third Xiangya Hospital of Central South University, 410013 Changsha, Hunan, People’s Republic of China

^2^ Department of Medicine, Third Xiangya Hospital of Central South University, 410013 Changsha, Hunan, People’s Republic of China

**Supplementary Methods**

**Cell culture**

HeLa cells were cultured in Dulbecco’s modified Eagle’s medium (DMEM; Gibco, CA, USA) and supplemented with 10% fetal bovine serum (FBS; Biological Industries, Israel) and 1% antibiotics (100 U/mL of penicillin and 100 mg/mL of streptomycin; Life Technologies, NY, USA). HL-1 cells were cultured in Clay-comb medium (Sigma Aldrich, MO, USA) supplemented with 10% FBS, 1% antibiotics (Life Technologies), 0.1 mM norepinephrine (Sigma Aldrich), and 2 mM glutamine (Sigma Aldrich). All cells used in this study were maintained in a 5% CO_2_ cell culture incubator at 37 ℃.

**Viral plaque assay**

The culture medium was serially diluted and added to HeLa cells in six-well plates (1 × 10^6^ cells/well). After co-culture for 1 h, the plates were washed twice with PBS, covered with 2% soft agar medium, and placed upside down in an incubator to continue incubation for 72 h. Viral plaques were counted using the VisionWorks system (Analytik Jena AG, Jena, Germany). Viral titers were calculated as plaque formation units per milliliter (pfu/ml). All assays were performed at least three times. All the assays were conducted at least three times.

**Immunofluorescence (IF) assay**

The obtained paraffin sections or the prepared cell crawls were processed for immunofluorescence staining. Briefly, for paraffin sections, the sections are dewaxed, dehydrated and boiled in Tris antigen retrieval buffer. Then, sections are incubated with anti-CVB3 (1:100) primary antibody overnight at 4°C. For cell crawl sections, fixation with 4% paraformaldehyde (10 min) and permeabilization with 0.5% Triton X-100 (10 min) was performed at room temperature. Afterwards, they were blocked with 5% goat serum for 2 h. Finally, the cell crawls were incubated overnight at 4°C with primary antibodies against FOXO1 (1:100). Subsequently, for both paraffin sections and cell crawls incubated with Alexa Fluor 594 or 488-labeled secondary antibodies (1:200) for 1 h at room temperature. DAPI (cell nuclear stain, final concentration 125 μg/ml) was added after 15 min of secondary antibody incubation. Finally, the samples were observed under a fluorescent microscope (Zeiss, Jena, Germany). Each procedure was repeated three times. The antibodies in this study are provided in *Supplementary Table S2*.

**Cytotoxicity and cell proliferation**

Calcein-AM (2 μM) and PI (4.5 μM) were added to the culture medium and incubated with HL-1 cells for 10 min at 37 °C with 5% CO_2_. Then, the samples were observed under fluorescence microscope (Zeiss). The results were obtained from at least three independent experiments.

**Western blotting**

Myocardial tissue blocks were placed in radioimmunoprecipitation assay (RIPA) buffer (Beyotime, Shanghai, China) supplemented with protease inhibitor (Roche), phosphatase inhibitor (Roche) and 1 mM phenylmethylsulfonyl fluoride using MagNA Lyser homogenizer (Roche) to grind the lysed tissue. The above operations were performed on ice. For cell, washed three times with phosphate-buffered saline (PBS) (Servicebio, Wuhan, China) and then lysed on ice with RIPA with a cocktail containing protease inhibitor and phosphatase inhibitor (Thermo Fisher, CA, USA). Proteins were separated by 8-12% sodium dodecyl sulfate-polyacrylamide gel electrophoresis (SDS-PAGE) and transferred to polyvinylidene fluoride membranes (PVDF) (Millipore, CA, USA). The membranes were closed with 5% skim milk for 1 h at room temperature, then incubated with primary antibody overnight at 4°C and secondary antibody for 1 h. The signals were visualized with an enhanced chemiluminescence (ECL) kit (Biosharp, Anhui, China), photographed and measured with the VisionWorks system (Analytik Jena AG). These results are representative of at least three independent experiments. The antibodies in this study are provided in *Supplementary Table S2*.

**Subcellular fractionation assay**

According to the instructions, removing the culture medium of adherent cultured cells, 5 mL of cooled 1× PBS rinsing solution was added to the 10-cm culture dish for washing twice, and the cells were scraped off. Next, the cells were transferred to centrifuge tubes and centrifuged at 800 × g at 4 °C for 10 min, the prepared cooled hypotonic buffer was added. The precipitate was resuspended, ice-bathed for 10 min, shaken for 10 s and mixed evenly. Finally, the suspension was centrifuged and the supernatant was the cytoplasm protein, which was transferred to clean cooled tubes. Then, 0.2 mL of lysis buffer was added to the precipitation, and centrifuged at 3000 × g at 4 °C for 10 min, the supernatant was the nuclear protein extract. Using Pierce BCA Protein Assay Kit to quantify the protein, and western blot was used to measure the expression of FOXO1 in nuclear and cytoplasm. The internal parameters for cell fractionations: LaminB1 for nuclear protein and β-Tubulin for cytoplasm protein. The antibodies in this study are provided in Supplementary Table S2.

**Dual-luciferase reporter assay**

Firstly, the Bim promoter region was searched in the NCBI database (http://www.ncbi.nlm.nih.gov/) and the Ensembl database (http://useast.ensembl.org/index.html). The results revealed a segment of -1900 to +350 bp from the transcription start site. The gene fragment was then cloned into the 5'Kpnl-3'SacI site of the pGL3-promoter vector (Promega, WI, USA). HL-1 cells were then inoculated on 24-well plates and co-transfected with Lipofectamine 3000 (Invitrogen) with plasmids of FOXO1 plasmids (si-FOXO1, FOXO1-WT and FOXO1-MT), Renilla luciferase and the Bim promoter. 48 h later, the cells were lysed according to the Dual-Luciferase® Report (Promega) instructions, cells were collected and cell lysates were analyzed using the EnVision Multilabel Plate Readers (PerkinElemer). All plasmids were verified by Sanger sequencing analysis before transfection.

**Chromatin immunoprecipitation (ChIP) assay**

HL-1 cells were sonicated to obtain optimal DNA fragment sizes of 200-1000 bp. Immunoprecipitation (IP) was performed overnight using anti-FOXO1 antibody (at 4°C). An unrelated rabbit IgG was used as a negative control. Finally, qRT-PCR was performed on purified input and immunoprecipitated DNA. The primer sequences for promoter region are listed in *Supplemental Table S1*.

**Co-immunoprecipitation (Co-IP) assay**

The total protein of the HL-1cells was extracted and lysed with IP lysis solution with Halt Protease and Phosphatase Inhibitor Cocktail (Thermo Fisher) on ice. After proper lysis for 15 min, the lysate was centrifuged at 12,000g for 30 min and the supernatant was separated. The total protein was then incubated with primary antibody at 4°C with spinning overnight. Then, the antigen-antibody complex was bound to magnetic beads at room temperature for 1 h and subsequently subjected to magnetic separation. Finally, the purified proteins obtained were subjected to Western blotting experiments. The antibodies in this study are provided in *Supplementary Table S2*.
